# A mechanistic study on the enhanced antihypertensive effects of a *Dendrobium officinale* compound combined with Western antihypertensive drugs in spontaneously hypertensive rats based on metabolomics and gut microbiota analysis

**DOI:** 10.3389/fcell.2026.1806395

**Published:** 2026-06-15

**Authors:** Renzhao Wu, Cheng Tong, Zedong Gong, Jing Wang, Jie Wang, Tuo Feng, Xiaomin Xue, Xiyu Mei, Zhongxiu Guo, Yue Wu, Shuwei Huang, Caicai Xi

**Affiliations:** 1 School of Basic Medical Sciences, Zhejiang Chinese Medical University, Hangzhou, China; 2 Zhejiang Academy of Traditional Chinese Medicine, Hangzhou, China; 3 Linping Campus, The Second Affiliated Hospital of Zhejiang University School of Medicine, Hangzhou, China; 4 Zhejiang Provincial Key Laboratory of Traditional Chinese Medicine for Pharmacodynamic Material Basis Research of Chinese Medicine, Hangzhou, China; 5 The First Affiliated Hospital of Zhejiang Chinese Medical University (Zhejiang Provincial Hospital of Chinese Medicine), Hangzhou, China; 6 Clinical Pharmacy Department, Hangzhou TCM Hospital Affiliated to Zhejiang Chinese Medical University, Hangzhou, China

**Keywords:** *Dendrobium officinale* compound, enhanced antihypertensive effects, gut microbiota, metabolomics, spontaneously hypertensive

## Abstract

**Background:**

In our previously published research, we reported that the combination of Dendrobium Officinale Compound (DOC) and antihypertensive medications (irbesartan and amlodipine besylate) significantly and stably regulates blood pressure in spontaneously hypertensive rats (SHRs). This study aims to further explore the unique mechanisms and pathway networks underlying the antihypertensive effects of this combination therapy at the molecular level, based on serum and ileocecal contents metabolomics as well as gut microbiota analysis.

**Methods:**

Utilizing UPLC-Q-TOF/MS and metabolomics techniques, we collected, analyzed, and integrated metabolite data to identify differential small molecule metabolites. High-throughput sequencing of the 16S rRNA gene was employed to characterize differences in gut microbiota composition among experimental animals subjected to distinct pharmacological interventions, and further elucidating the underlying antihypertensive mechanism network associated with the combined administration of DOC and conventional antihypertensive drugs.

**Results:**

Metabolomic analyses revealed substantial differences in the profiles of low-molecular-weight metabolites in both serum and ileocecal contents across all treatment groups compared with the model control group. The antihypertensive effects of medium- and high-dose DOC in SHRs were associated with differential metabolites including L-tyrosine, L-methionine, and L-arginine; in contrast, the enhanced antihypertensive effect observed upon combination therapy with DOC and Western antihypertensive drugs correlated with distinct metabolites such as pregnenolone, creatine, and L-phenylalanine. 16S rRNA gene high-throughput sequencing of ileocecal contents indicated that the relative abundance of Firmicutes increased by 8.73%, 0.84%, and 10.40% following low-, medium-, and high-dose DOC interventions, respectively, relative to the model group; however, combination therapy with DOC and Western antihypertensive drugs resulted in an 8.80% decrease in Firmicutes abundance compared with the model group. Conversely, the relative abundance of Bacteroidetes decreased by 7.60%, 3.31%, and 7.67% after low-, medium-, and high-dose DOC treatments, respectively, versus the model group; yet, combination therapy led to a 7.05% increase in Bacteroidetes abundance relative to the model group.

**Conclusion:**

Metabolomic and 16S rRNA sequencing analyses revealed that these effects may be linked to DOC’s specific modulation of serum amino acid profiles and remodeling of gut microbiota composition in PCAs. These findings provide new mechanistic insights into this traditional herb’s antihypertensive action.

## 1 Introduction

Hypertension is the most common chronic cardiovascular disorder, characterized primarily by persistently elevated arterial blood pressure. It may lead to multi-organ damage and complications involving multiple physiological systems. In 2003, the World Health Organization identified hypertension as a leading global risk factor for the incidence and mortality of various diseases (Kjeldsen, 2018), a status that remains valid to date (Kuneinen et al., 2024). Researchers have projected that the global population of individuals with hypertension will exceed 1.5 billion, with both the risk of developing the condition and its associated health burden increasing significantly with age (Ma et al., 2019). A substantial body of research evidence indicates that, in clinical practice, only a small proportion of hypertensive patients achieve blood pressure levels below the thresholds recommended by clinical guidelines, and this proportion may be even lower in developing countries (Kearney et al., 2004). Furthermore, approximately 30% of hypertensive patients remain uncontrolled despite the use of three or more antihypertensive medications, a condition commonly referred to as resistant hypertension (Egan, 2015). The epidemiological characteristics of hypertension have been established against the backdrop of extensive research and widespread clinical application of various antihypertensive drugs. Therefore, the limitations associated with current antihypertensive medications warrant careful consideration by researchers. Meanwhile, exploring innovative therapeutic approaches and novel targets for hypertension management will become an urgent necessity in improving the global prevention and control of this condition.

Due to the pharmacological characteristics of traditional Chinese medicine (TCM)—namely, its multi-component and multi-target nature—it can simultaneously delay disease progression and modulate immune function, metabolism, and other physiological processes, thereby offering advantages over single-agent Western pharmaceuticals. The growing interest in combining TCM and Western medicines for hypertension management stems from persistent challenges in modern hypertension care—such as therapeutic plateaus and suboptimal long-term control—as well as the distinctive holistic regulatory strengths of TCM. A meta-analysis incorporating 29 randomized controlled trials (RCTs) involving 2,623 patients with primary hypertension demonstrated that, compared with Western medicine monotherapy, the combination therapy group achieved a significantly higher overall clinical efficacy rate (risk ratio [RR] = 1.23; 95% confidence interval [CI], 1.17–1.30) and a markedly greater marked-effect rate (RR = 1.66). In terms of blood pressure reduction, the combination group exhibited an additional mean decrease of 7.91 mmHg in systolic blood pressure and 5.46 mmHg in diastolic blood pressure relative to the Western medicine monotherapy group. Regarding safety, no serious adverse events were reported in the combination therapy group (Mohammed et al., 2023). Moreover, combining TCM with Western antihypertensive therapy not only enhances blood pressure-lowering efficacy but also effectively reduces serum lipid levels, thereby further decreasing the incidence of cardiovascular events in patients (Francis et al., 2024). That is to say, the combined use of traditional Chinese medicine (TCM) and Western antihypertensive agents represents an integrative strategy that leverages TCM’s unique advantages—namely, its holistic regulatory effects and multi-target pharmacological actions—to achieve enhanced complementarity with modern antihypertensive drugs. This approach holds promise for optimizing blood pressure control in clinical practice, improving patients’ quality of life, and reducing the risk of cardiovascular events.

*Dendrobium officinale* Kimura et Migo, a species of the Orchidaceae family, has been traditionally used in Chinese medicine for thousands of years. Its dried or fresh stems are commonly prepared as decoctions or herbal teas to alleviate various conditions, including chronic pharyngitis, insomnia, irritability, and hot flashes (Zhang et al., 2023). Recent studies have demonstrated that D. officinale ultrafine powder exerts significant antihypertensive effects by modulating gut microbiota composition in rat models of metabolic hypertension, increasing levels of short-chain fatty acids (SCFAs) in both serum and intestinal tissues, activating the SCFA-GPCR 43/41 signaling pathway, and consequently improving vascular endothelial function (Li et al., 2021). In our previous studies, we developed Dendrobium officinale Compound (DOC), primarily composed of *Dendrobium officinale* and *Paeonia lactiflora*, which exhibited stable and sustained antihypertensive effects in spontaneously hypertensive rats (SHRs). Furthermore, when DOC was co-administered with two conventional antihypertensive drugs—irbesartan and amlodipine besylate—the therapeutic efficacy was enhanced. Through serum analysis of SHRs following intervention, five chemical monomers were identified as potentially closely associated with the antihypertensive effects (Wu et al., 2025). However, the precise underlying mechanisms remain to be elucidated.

Metabolomics, which provides a comprehensive characterization of metabolites in biological systems, has been widely applied in disease diagnosis, the exploration of novel drug targets, and the investigation of pharmacological actions and mechanisms. However, due to the inherent limitations in detection coverage, single-omics approaches often fail to systematically elucidate the pharmacological effects of complex formulations, such as traditional Chinese medicine prescriptions. The gut microbiota represents the most diverse and abundant microbial community within the human body. It is now widely recognized that metabolites produced by the gut microbiota significantly influence the host’s metabolic profile, thereby triggering a cascade of metabolic alterations (Lindsay et al., 2020). These changes hold substantial value for understanding the pathogenesis of various diseases and the pharmacological mechanisms of therapeutic agents. In recent years, substantial evidence has demonstrated that hypertensive individuals exhibit gut microbiota dysbiosis and impaired intestinal barrier function (Yang et al., 2023). Notably, 16S rRNA sequencing enables resolution of alterations in gut microbial community structure, whereas untargeted metabolomics provides a comprehensive profiling of host metabolic signatures. Integrating these two complementary approaches allows construction of an integrative association network linking “gut microbiota–host metabolism–blood pressure regulation,” thereby offering system-level evidence to elucidate the multi-target enhanced mechanisms underlying the combined use of TCM formulae and Western pharmaceuticals.

This study aims to build upon previous research by integrating 16S rRNA sequencing and metabolomics, and employing correlation analysis to elucidate the associations between gut bacterial taxa and differential metabolites. The underlying antihypertensive mechanisms of the DOC in combination with two Western antihypertensive drugs will be further investigated in SHRs, thereby providing a scientific reference for the clinical application of combining DOC with conventional medications in the treatment of essential hypertension.

## 2 Materials and methods

Metabolomics is a comprehensive analytical technique for the qualitative and quantitative profiling of low-molecular-weight metabolites (molecular weight <1,500 Da) in biological samples—including serum, tissues, and ileocecal contents—aimed at elucidating characteristic metabolic responses of organisms under physiological or pathological perturbations. In this study, untargeted metabolomic analysis was performed on rat serum and ileocecal contents using ultra-performance liquid chromatography coupled with quadrupole time-of-flight mass spectrometry (UPLC-Q-TOF/MS), enabling the identification of differentially abundant metabolites and subsequent enrichment analysis of associated metabolic pathways.

16S rRNA gene sequencing is currently one of the most widely employed methods for characterizing microbial community composition and structure. This approach involves amplifying hypervariable regions of the bacterial 16S ribosomal RNA gene and coupling the amplification with high-throughput sequencing to enable taxonomic classification and diversity analysis of gut microbiota. In this study, we applied this methodology to analyze the intestinal microbiota present in ileocecal contents of rats, thereby evaluating the impact of distinct drug administration regimens on gut microbial ecology (Supplementary Figure 1).

### 2.1 Instruments and materials

Instrumentation: BP-98A non-invasive tail-cuff blood pressure monitor (Softron Co., Ltd., Japan); DK-450B electric thermostatic water bath (Shanghai Sension Experimental Instruments Co., Ltd.); SCIEX X-500R quadrupole time-of-flight mass spectrometer equipped with a TurboIonSpray ion source (AB SCIEX, USA); Waters ACQUITY I-Class Plus UPLC system (Waters Corporation, USA); Thermo ST40R refrigerated high-speed centrifuge (Thermo Fisher Scientific, USA); CentriVap acid-resistant centrifugal concentrator (LABCONCO, USA); MM400 cryogenic ball mill (Retsch GmbH, Germany).

Experimental drug: Dendrobium Officinale Compound (DOC) (specification: 1.5 g/tablet), with each Gram containing approximately 0.777 g of crude herbal mixture, batch number: 201905002, provided by Zhejiang Academy of Traditional Chinese Medicine. The DOC consists of *Dendrobium officinale* and *Paeonia lactiflora* in a ratio of 4:3, processed into powder, combined with excipients, and compressed into tablets. Irbesartan tablets (specification: 0.15 g/tablet, batch number: 20042611) were provided by Hanhui Pharmaceutical Co., Ltd.; Amlodipine Besylate Tablets (specification: 5 mg/tablet, batch number: 191251304) were provided by Suzhou Dongrui Pharmaceutical Co., Ltd.

Experimental reagents: methanol, acetonitrile, and formic acid (Merck, Germany); Milli-Q ultrapure water (Millipore, USA).

### 2.2 Experimental animals

Six 2-month-old female Wistar rats, weighing 236.1 ± 17.96 g, were obtained from Beijing Vital River Laboratory Animal Technology Co., Ltd. The production license number for experimental animals is SCXK (Zhejiang) 2020-0002, and the certificate of conformity number is 20201105Aazz0600067213. Forty-two 2-month-old female SHRs, weighing 165.63 ± 7.98 g, were also provided by Beijing Vital River Laboratory Animal Technology Co., Ltd. The production license number for experimental animals is SCXK (Beijing) 2016-0006, and the certificate of conformity number is 110011201108553221. This study was approved by the Animal Welfare and Ethics Committee of Zhejiang Academy of Traditional Chinese Medicine (Approval No.: [2022]054).

All rats were housed at the Experimental Animal Center of Zhejiang Academy of Traditional Chinese Medicine (Approval No.: SCXK (Zhejiang) 2019-0010). The environmental temperature was maintained at (25 ± 2)°C, and relative humidity was kept between 55% and 60%. The rats were fed a standard pelleted diet and maintained under a 12-h light–12-h dark cycle. After 1 week of acclimatization, the experiments were initiated.

Euthanasia of rats: Anesthesia was induced in an induction chamber by delivering 3% isoflurane in 100% oxygen at a flow rate of 1 L/min. Upon successful induction, anesthesia was maintained with 1%–2% isoflurane. Blood and ileocecal contents were then collected. Following completion of sample collection, euthanasia was performed by administering 3%–5% isoflurane.

Six Wistar rats were assigned to the normal control group (Normal), and 42 SHRs were randomly divided into seven groups with six rats in each group: model group (Model), low-dose DOC mixture group (LF), medium-dose DOC group (MF), high-dose DOC group (HF), Irbesartan group (IRB), combination therapy of Irbesartan and Amlodipine Besylate group (IAC), and DOC combined with dual antihypertensive drugs group (FIAC). The gavage volume for all groups was 2 mL/100 g body weight, administered once daily for six consecutive weeks. Rats in the LF, MF, and HF groups received the crude herbal formula at doses of 0.7 g/kg, 1.4 g/kg, and 2.8 g/kg, respectively. The IRB group received irbesartan at 15 mg/kg, the IAC group received a combination of irbesartan (15 mg/kg) and amlodipine (0.5 mg/kg), and the FIAC group received the herbal formula (2.8 g/kg) plus irbesartan (15 mg/kg) and amlodipine (0.5 mg/kg). The Normal and Model groups were administered an equivalent volume of physiological saline. General conditions of the rats, including appearance, behavior, mental status, food and water intake, body weight changes, and general activity, were observed throughout the study. Blood pressure was measured on two different days before treatment initiation, and the average value was recorded as the baseline. After the first administration and every 2 weeks thereafter, blood pressure was measured at 3 h and 24 h post-dosing. Body weight was recorded weekly. At the end of the experiment, following the final blood pressure measurement, rats were anesthetized and blood samples were collected via abdominal aorta puncture. The blood was allowed to stand at room temperature for 2 h, then centrifuged at 3,000 r/min for 10 min at 4 °C. Supernatants (200 μL) were aliquoted and stored at −80 °C for subsequent metabolomics analysis. Under sterile conditions, ileocecal contents were collected: one portion was immediately frozen in dry ice and sent to Meigu Gene Technology Co., Ltd. (Guangdong, China) for DNA extraction and sequencing; another portion was stored at −80 °C for later metabolomics analysis. Hearts were harvested and weighed to calculate the heart-to-body weight ratio (heart weight/body weight). Cardiac tissues were sectioned, placed in cryotubes, rapidly frozen in liquid nitrogen, and subsequently stored at −80 °C for future use.

### 2.3 Blood pressure measurement in rats

Blood pressure was measured in rats at the following time points: prior to drug administration; at 3 h and 24 h after the first dose; and at 3 h and 24 h after dosing on Week 2, Week 4, and Week 6. During blood pressure measurement, the rats were heated on the iron plate of a 38.3 °C water bath. To avoid excessive heating caused by individual differences in each group of rats, the first heating time was 4 min, if the blood pressure data could not be obtained, the number of heating sessions was increased, and the second heating time was 2 min. The data were obtained on the computer through the BP-98A-type noninvasive tail artery blood pressure measuring instrument supporting software. The blood pressure of each rat was measured 6–10 times, and the mean value was used as the measurement value of that rat.

### 2.4 Sample preparation

Serum Sample Preparation: Serum samples (200 μL) were thawed, and 600 μL of methanol/acetonitrile (1:1, v/v) containing the internal standard L-2-chlorophenylalanine at a concentration of 10 μmol/L was added. The mixture was vortexed for 60 s, followed by centrifugation at 14,000 × g for 20 min at 4 °C. The supernatant was transferred to a 1.5 mL centrifuge tube and subjected to freeze-drying. The dried sample residue was reconstituted with a solvent mixture consisting of acetonitrile/methanol (80:20, v/v) and ultrapure water in a 1:1 (v/v) ratio. The mixture was vortexed for 1–2 min, then centrifuged again at 14,000 × g for 20 min at 4 °C. The resulting supernatant was transferred to an autosampler vial for analysis. A quality control (QC) sample, prepared by pooling equal aliquots from all study samples, was injected after every six analytical samples throughout the sequence.

Ileocecal content samples preparation from the ileocecal region: In this study, the ileocecal contents referred to are primarily those collected from the ileocecal junction. A 10 mg solid sample was excised and precisely weighed in a centrifuge tube. Subsequently, 20 μL of ultrapure water and 180 μL of acetonitrile/methanol (8:2, v/v) solution containing the internal standard L-2-chlorophenylalanine (at a concentration of 10 μmol/L) were added. The mixture was homogenized and then centrifuged at 14,000 rpm for 20 min at 4 °C. The resulting supernatant was transferred to a new centrifuge tube and lyophilized. The dried sample powder was reconstituted with a 1:1 (v/v) mixture of acetonitrile/methanol (80/20, v/v) and ultrapure water, vortexed for 1–2 min, and centrifuged again at 13,500 g for 20 min at 4 °C. The final supernatant was transferred into an autosampler vial for analysis. A quality control (QC) sample was analyzed after every six experimental samples to ensure analytical consistency.

### 2.5 Chromatographic and mass spectrometric conditions

Chromatographic conditions: ACQUITY UPLC® BEH C18 column (100 × 2.1 mm, 1.7 μm), flow rate: 0.3 mL/min; autosampler temperature: 8 °C; column temperature: 50 °C; injection volume: 2 μL. The mobile phase consisted of 0.1% formic acid in acetonitrile (A) and 0.1% formic acid in water (B), with gradient elution.

Mass spectrometry conditions: A Waters quadrupole time-of-flight mass spectrometer (Q-TOF/MS) was employed. The time-of-flight mass spectrometer was equipped with a TurboIonSpray ion source and operated in both positive and negative electrospray ionization (ESI) modes. Specific parameters were as follows: nebulizer gas (GS1) and heater gas (GS2) were set to 55 psi, curtain gas (CUR) was maintained at 35 psi, ion source temperature was 600 °C, and the ion spray voltage (ISVF) was set to +5500 V for positive mode and −4500 V for negative mode. The TOF/MS scan range was from m/z 50–1,000 Da.

### 2.6 High-throughput sequencing of the 16S rRNA gene

Individual samples from the control group, model group, and drug-treated group were subjected to 16S rRNA sequencing. Microbial DNA was extracted from each ileocecal sample using a magnetic bead-based fecal DNA extraction kit according to the manufacturer’s instructions. The integrity and purity of the extracted DNA were assessed by 1% agarose gel electrophoresis, while the DNA concentration and purity were determined using a NanoDrop One (Thermo Fisher Scientific) spectrophotometer. PCR amplification of the V3–V4 hypervariable regions of the bacterial 16S rRNA gene was performed using specific primers (338F: 5′-ACTCCTACGGGAGGCAGCA-3′and 806R: 5′-GGACTACHVGGGTWTCTAAT-3′) and Premix Taq (TaKaRa). The PCR products were quantified and compared using Gene Tools Analysis Software (Version 4.03.05.0, SynGene), and equal amounts of each product were pooled based on their concentrations. The mixed PCR products were purified using the E.Z.N.A.® Gel Extraction Kit, and the target DNA fragments were eluted with TE buffer. Library construction was carried out following the standard protocol of the NEBNext® Ultra™ DNA Library Prep Kit for Illumina®. The constructed libraries were then sequenced on the HiSeq high-throughput sequencing platform. Subsequent bioinformatic analyses included sequence processing and taxonomic annotation. Alpha and beta diversity analyses were conducted to evaluate microbial community differences among samples. Additionally, LEfSe (Linear Discriminant Analysis Effect Size) analysis was employed to identify differentially abundant microbial taxa across the experimental groups.

### 2.7 Statistical methods

Data were statistically analyzed using SPSS 25.0 software. For normally distributed data, one-way analysis of variance (ANOVA), LSD-t and Dunnett’s T3 tests, and independent samples t-tests were employed. For non-normally distributed data, the rank-sum test was applied. Data are presented as mean ± standard deviation (x ± s), with P < 0.05 indicating statistical significance. Rat serum and tissue samples underwent data acquisition via UPLC-Q-TOF/MS. Raw data were converted to mzXML format using MSConvert software. The mzXML data were imported into the XCMSplus offline workstation, where peak extraction was performed using the multigroup job function. MetDNA was used for differential metabolite identification. Metabolites were analyzed via OPLS-DA using SIMCA-P 14.1, with metabolites exhibiting VIP >1 selected. These metabolites were then imported into MetaboAnalyst 6.0 for pathway analysis, ultimately yielding differential metabolic pathways.

## 3 Results

### 3.1 The hypotensive efficacy of different concentrations of DOC and combined use of Chinese and Western medicines in SHRs

Prior to administration, the systolic blood pressure in the normal group was significantly lower than that in the model group (P < 0.01), while no statistically significant differences were observed between the treatment groups and the model group (Table 1). Comprehensive analysis of the entire 6-week intervention period revealed that DOC at different concentrations exerted varying degrees of therapeutic effects on blood pressure in SHRs (P < 0.05). Among the treatment groups, the HF and MF groups demonstrated significantly greater antihypertensive efficacy compared to the LF group. The blood pressure reduction in the LF group showed a marked decline at the 24th hour relative to the 3rd hour in each dosing cycle, whereas the HF group maintained a relatively higher level of blood pressure reduction throughout, with no evident rebound at the 24th hour (Supplementary Table 1). Overall, although the LF group exhibited significant antihypertensive effects, the response was less stable. In contrast, the HF group achieved the most optimal and consistent blood pressure control. The magnitude of blood pressure reduction in the LF group was significantly weaker than that in the IRB group (p < 0.05), whereas the antihypertensive effects of the MF and HF groups were comparable to those of the IRB group, with no statistically significant differences, indicating that medium- and high-concentration DOC regimens achieve therapeutic efficacy similar to that of irbesartan. Furthermore, the combination of irbesartan and amlodipine besylate also demonstrated favorable antihypertensive effects, comparable to those of high-concentration DOC. Notably, when either of these two conventional antihypertensive drugs was combined with high-concentration DOC, the antihypertensive effect was further enhanced (p < 0.01), making these combination regimens the most effective interventions in this study.

**TABLE 1:** Effects of different concentrations of DOC, as well as high-concentration DOC in combination with western medicine, on the antihypertensive efficacy in sHRs.

Administration time	Normal	Model	IRB	LF	MF	HF	IAC	FIAC
Prior to administration	115.90 ± 3.17	184.19 ± 2.82^**^	185.60 ± 2.70	183.49 ± 2.81	184.06 ± 3.44	182.64 ± 1.97	183.33 ± 4.84	183.13 ± 2.00
First administration at 3 h post-dose	117.32 ± 5.09	182.60 ± 4.50^**^	162.02 ± 6.21△△	172.38 ± 0.90△◆◆▲	162.42 ± 5.23△△	159.05 ± 5.13△△◇◇	159.13 ± 5.90△△◆◆	139.72 ± 3.05△△##
First administration at 24 h post-dose	116.43 ± 6.41	184.81 ± 2.82^**^	169.12 ± 2.27△△◆	181.22 ± 2.23△##◆◆▲▲	168.49 ± 3.82△△	165.55 ± 3.07△△#◇◇	163.02 ± 5.87△△◆◆	147.10 ± 3.14△△##
At 3 h on the 2nd week of dosing	117.12 ± 4.80	183.96 ± 3.76^**^	163.83 ± 2.80△△	174.02 ± 5.71△△##◆◆▲▲	162.81 ± 3.08△△	161.02 ± 2.04△△◇◇	161.10 ± 2.83△△◆◆	142.07 ± 6.67△△##
At 24 h on the 2nd week of dosing	111.44 ± 10.21	184.63 ± 2.60^**^	163.48 ± 6.00△△	182.26 ± 2.64##◆◆▲▲	167.04 ± 2.50△△	163.62 ± 3.02△△◇◇	160.96 ± 3.08△△◆◆	147.21 ± 3.68△△##
At 3 h on the 4th week of dosing	114.05 ± 3.04	183.45 ± 2.23^**^	162.67 ± 2.78△△	172.72 ± 1.53△△##◆◆▲▲	162.45 ± 2.38△△	160.85 ± 1.70△△◇◇	160.00 ± 1.84△△◆◆	139.60 ± 3.57△△##
At 24 h on the 4th week of dosing	114.03 ± 5.73	186.10 ± 1.82^**^	164.13 ± 1.41△△	181.90 ± 2.28△△##◆◆▲▲	166.10 ± 3.06△△	164.76 ± 0.85△△◇◇	161.52 ± 1.95△△◆◆	148.93 ± 4.10△△##
At 3 h on the 6th week of dosing	115.79 ± 5.55	184.86 ± 2.16^**^	163.52 ± 3.17△△	171.93 ± 2.13△△##◆◆▲▲	164.82 ± 2.70△△	163.02 ± 4.49△△◇◇	160.78 ± 1.72△△◆◆	138.31 ± 3.08△△##
At 24 h on the 6th week of dosing	115.67 ± 9.03	184.42 ± 2.43^**^	165.26 ± 4.10△△	181.52 ± 1.88##◆◆▲▲	165.92 ± 0.94△△	165.37 ± 0.81△△◇◇	164.18 ± 2.67△△◆◆	149.11 ± 2.79△△##

Normal: Wistar rat group; Model: SHRs model group; IRB: treatment group administered with Irbesartan; LF: treatment group administered with 0.7 g/kg DOC (low concentration); MF: treatment group administered with 1.4 g/kg DOC (medium concentration); HF: treatment group administered with 2.8 g/kg DOC (high concentration); IAC: treatment group receiving combination therapy of Irbesartan and Amlodipine Besylate; FIAC: treatment group receiving high-dose DOC in combination with the two Western antihypertensive drugs. Comparison between the Model group and the Normal group:**: P < 0.01. Comparisons among treatment groups and the Model group: △:P < 0.05, △△: P <0.01. Comparisons among treatment groups and the IRB group: ##: P < 0.01. Comparisons among treatment groups and the HF group: ◆◆: P < 0.01. Comparisons among treatment groups and the MF group: ▲: P< 0.05, ▲▲: P< 0.01. Comparisons among treatment groups and the FIAC group: ◇◇: P < 0.01.

### 3.2 Serum and ileocecal contents metabolomics results

Serum and ileocecal content samples were analyzed using UPLC-Q-TOF/MS. Raw data were converted to mzXML format using MSConvert software, and the mzXML files were subsequently imported into the XCMSplus offline platform. Peak extraction was performed using the multigroup job function, followed by differential metabolite identification with MetDNA. Ultimately, 34 metabolites were identified in serum under positive ion mode (Table 2), 11 metabolites in serum under negative ion mode (Table 3), 60 metabolites in ileocecal content samples under positive ion mode (Table 4), and 15 metabolites in ileocecal content samples under negative ion mode (Table 5).

**TABLE 2:** Serum POS data MetDNA identification results.

No.	Metabolite name	Time (min)	Molecular formula	Quant mz
1	L-Carnitine	0.84	C_7_H_15_NO_3_	162.1127
2	Citronellate	14.24	C_10_H_18_O_2_	171.1379
3	L-Tyrosine	0.97	C_9_H_11_NO_3_	182.081
4	Phenylacetonitrile	2.56	C_8_H_7_N	118.0651
5	O-Acetylcarnitine	0.91	C_9_H_17_NO_4_	204.1229
6	Spermidine	0.74	C_7_H_19_N_3_	146.1652
7	Abrine	3.06	C_12_H_14_N_2_O_2_	219.1131
8	L-Octanoylcarnitine	4.55	C_15_H_29_NO_4_	288.2169
9	L-Histidine	0.76	C_6_H_9_N_3_O_2_	156.0768
10	Urea	0.86	CH_4_N_2_O	61.0395
11	L-Methionine	0.95	C_5_H_11_NO_2_S	150.0582
12	L-Arginine	0.8	C_6_H_14_N_4_O_2_	175.1191
13	11-Dehydrocorticosterone	4.88	C_21_H_28_O_4_	345.2063
14	Serotonin	0.98	C_10_H_12_N_2_O	177.1023
15	Deoxycytidine	0.88	C_9_H_13_N_3_O_4_	228.0983
16	11-Deoxycortisol	5.94	C_21_H_30_O_4_	347.2215
17	4-Methyl-2-oxopentanoate	4.18	C_6_H_10_O_3_	131.0686
18	Gamma-Butyrolactone	1.28	C_4_H_6_O_2_	87.044
19	Urate	0.9	C_5_H_4_N_4_O_3_	169.0356
20	Progesterone	6.91	C_21_H_30_O_2_	315.2319
21	Creatine	0.86	C_4_H_9_N_3_O_2_	132.0766
22	N-Methylhydantoin	0.9	C_4_H_6_N_2_O_2_	115.0508
23	L-Palmitoylcarnitine	8.25	C_23_H_45_NO_4_	400.3419
24	L-Phenylalanine	1.96	C_9_H_11_NO_2_	166.0862
25	Cytosine	0.89	C_4_H_5_N_3_O	112.0506
26	L-Proline	0.86	C_5_H_9_NO_2_	116.0706
27	2-Quinolinecarboxylic acid	2.96	C_10_H_7_NO_2_	174.0553
28	L-Pipecolate	0.77	C_6_H_11_NO_2_	130.0862
29	L-Leucine	1.35	C_6_H_13_NO_2_	132.1018
30	5-Aminopentanoate	0.87	C_5_H_11_NO_2_	118.0862
31	Tetrahydrocorticosterone	5.84	C_21_H_34_O_4_	351.253
32	3alpha,20alpha,21-Trihydroxy-5beta-pregnan-11-one	5.43	C_21_H_34_O_4_	351.2529
33	11beta,21-Dihydroxy-5beta- pregnane-3,20-dione	5.41	C_21_H_32_O_4_	349.2376
34	Pregnenolone	6.26	C_21_H_32_O_2_	317.2473

**TABLE 3:** Serum NEG data MetDNA identification results.

NO.	Metabolite name	Time (min)	Molecular formula	Quant mz
1	L-Phenylalanine	1.97	C_9_H_11_NO_2_	164.0715
2	Taurine	0.82	C_2_H_7_NO_3_S	124.0073
3	L-Histidine	0.78	C_6_H_9_N_3_O_2_	154.0623
4	Ascorbate	0.91	C_6_H_8_O_6_	175.0252
5	Succinate	0.97	C_4_H_6_O4	117.0196
6	Pseudouridine	0.89	C_9_H_12_N_2_O_6_	243.0627
7	Citrate	0.93	C_6_H_8_O7	191.0197
8	Hyodeoxycholate	6.74	C_24_H_40_O_4_	391.2856
9	Chenodeoxycholate	5.71	C_24_H_40_O_4_	391.2855
10	Glycerone	0.98	C_3_H_6_O _3_	89.0239
11	D-Mannose	0.81	C_6_H_12_O _6_	179.0562

**TABLE 4:** Ileocecal contents POS data MetDNA identification results.

No.	Metabolite name	Tim (min)	Molecular formula	Quant mz
1	Urocanate	0.87	C_6_H_6_N_2_O_2_	139.0501
2	5-Acetamidopentanoate	0.98	C_7_H_13_NO_3_	160.0968
3	Benzyl 2-methyl-3-oxobutanoate	3.28	C_12_H_14_O_3_	207.1019
4	Alpha-Tocopherol	11.51	C_29_H_50_O_2_	431.3875
5	Harman	3.15	C_12_H_10_N_2_	183.0921
6	L-Tyrosine	0.94	C_9_H_11_NO_3_	182.0812
7	Sinapyl alcohol	3.65	C_11_H_14_O_4_	211.0943
8	Androstenediol	5.23	C_19_H_30_O_2_	291.2323
9	Pregnanediol	11.61	C_21_H_36_O_2_	321.2788
10	L-Proline	0.84	C_5_H_9_NO_2_	116.0706
11	Apigenin 7-O-beta-D-glucoside	3.32	C_21_H_20_O_10_	433.1138
12	Ergosta-5,7,22,24(28)-tetraen-3beta-ol	11.54	C_28_H_42_O	395.3309
13	2-Aminobut-2-enoate	0.84	C_4_H_7_NO_2_	102.0552
14	Lactose	0.84	C_12_H_22_O_11_	343.1239
15	Daidzin	3.04	C_21_H_20_O_9_	417.1194
16	L-Valine	0.84	C_5_H_11_NO_2_	118.0862
17	L-Carnitine	0.81	C_7_H_15_NO_3_	162.1125
18	Cortol	4.26	C_21_H_36_O_5_	369.2631
19	L-Palmitoylcarnitine	7.55	C_23_H_45_NO_4_	400.3419
20	2-Quinolinecarboxylic acid	2.83	C_10_H_7_NO_2_	174.0552
21	Hypoxanthine	0.91	C_5_H_4_N_4_O	137.0458
22	Nicotinamide	0.94	C_6_H_6_N_2_O	123.055
23	Creatine	0.82	C_4_H_9_N_3_O_2_	132.0767
24	L-Phenylalanine	1.98	C_9_H_11_NO_2_	166.0864
25	L-Leucine	0.97	C_6_H_13_NO_2_	132.102
26	Cytosine	0.84	C_4_H_5_N_3_O	112.0506
27	Glycitein	3.89	C_16_H_12_O_5_	285.0759
28	Chrysin	3.83	C_15_H_10_O_4_	255.0654
29	Pregnenolone	7.54	C_21_H_32_O_2_	317.2473
30	20-Hydroxy-3-oxopregn-4-en-21-al	9.25	C_21_H_30_O_3_	331.2244
31	N-Methyl-L-glutamate	0.89	C_6_H_11_NO_4_	162.0763
32	Picolinic acid	0.92	C_6_H_5_NO_2_	124.0394
33	4,4-Dimethyl-5alpha-cholesta-8,14,24-trien-3beta-ol	9.14	C_29_H_46_O	411.3617
34	D-Phenylalanine	1	C_9_H_11_NO_2_	166.0864
35	Apigenin	4.19	C_15_H_10_O_5_	271.0605
36	(24R,24(1)R)-Fucosterol epoxide	13.34	C_29_H_48_O_2_	429.3723
37	17alpha,21-Dihydroxy-5beta- pregnane-3,11,20-trione	4.11	C_21_H_30_O_5_	363.2172
38	Ergosta-5,7,22,24(28)-tetraen-3beta-ol	10.03	C_28_H_42_O	395.3312
39	Urocortisol	3.19	C_21_H_34_O_5_	367.2472
40	7alpha-Hydroxydehydroepiandrosterone	4.14	C_19_H_28_O_3_	305.2112
41	Cortolone	3.79	C_21_H_34_O_5_	367.2482
42	3alpha,7alpha,12alpha-Trihydroxy-5beta-cholestane	10.52	C_27_H_48_O_3_	421.3662
43	21-Hydroxypregnenolone	6.25	C_21_H_32_O_3_	333.2423
44	Allotetrahydrodeoxycorticosterone	9.14	C_21_H_34_O_3_	335.2577
45	5alpha-Pregnane-3,20-dione	8.88	C_21_H_32_O_2_	317.2478
46	Tetrahydroxy-5beta-cholestan	9.23	C_27_H_48_O_4_	437.3622
47	5alpha-Cholesta-7,24-dien-3beta-ol	10.92	C_27_H_44_O	385.3474
48	20alpha,22beta-Dihydroxycholesterol	11.67	C_27_H_46_O_3_	419.3524
49	20alpha-Hydroxycholesterol	8.86	C_27_H_46_O_2_	403.3569
50	11beta,17alpha,21-Trihydroxypregnenolone	3.29	C_21_H_32_O_5_	365.2321
51	11beta,17alpha,21-Trihydroxy-5beta-pregnane-3,20-dione	4.2	C_21_H_32_O_5_	365.2323
52	Corticosterone	5.14	C_21_H_30_O_4_	347.2218
53	5alpha-Ergosta-7,22-diene-3beta,5-diol	9.31	C_28_H_46_O_2_	415.357
54	3alpha,7alpha-Dihydroxy-5beta- cholestanate	9.63	C_27_H_46_O_4_	435.3468
55	22alpha-Hydroxy-campest-4-en-3-one	9.99	C_28_H_46_O_2_	415.3573
56	17alpha,21-Dihydroxypregnenolone	3.82	C_21_H_32_O_4_	349.2374
57	(25S)-26-Hydroxycholest-4-en-3-one	11.7	C_27_H_44_O_2_	401.3409
58	17alpha-Hydroxypregnenolone	4.05	C_21_H_32_O_3_	333.2422
59	21-Hydroxy-5beta-pregnane-3,11,20-trione	4.38	C_21_H_30_O_4_	347.2222
60	(25R)-26-Hydroxycholest-4-en-3-one	8.86	C_27_H_44_O_2_	401.3418

**TABLE 5:** Ileocecal contents NEG data MetDNA identification results.

No.	Metabolite name	Time (min)	Molecular formula	Quant mz
1	L-Glutamate	0.83	C_5_H_9_NO_4_	146.0459
2	Succinate	0.98	C_4_H_6_O4	117.0194
3	Tetrahydrocorticosterone	6.64	C_21_H_34_O_4_	349.2389
4	Oleanolic acid	9.43	C_30_H_48_O_3_	455.3532
5	Hypoxanthine	0.9	C_5_H_4_N_4_O	135.0312
6	16-Hydroxypalmitate	7.5	C_16_H_32_O_3_	271.2287
7	Taurine	0.83	C_2_H_7_NO_3_S	124.0075
8	N-Acetylneuraminate	0.85	C_11_H_19_NO_9_	308.0993
9	Isolithocholate	7.95	C_24_H_40_O_3_	375.2904
10	Lithocholic acid	6.86	C_24_H_40_O_3_	375.2904
11	Glycerone	0.98	C_3_H_6_O3	89.0243
12	3alpha,7alpha-Dihydroxy- 12-oxo-5beta-cholanate	4.63	C_24_H_38_O_5_	405.2646
13	3alpha,20alpha,21-Trihydroxy-5beta-pregnan-11-one	4.72	C_21_H_34_O_4_	349.2381
14	Cholic acid	4.4	C_24_H_40_O5	407.2799
15	3alpha,7alpha,12beta-Trihydroxy-5beta-cholanate	4.85	C_24_H_40_O_5_	407.2798

### 3.3 Serum of SHRs and principal component analysis (PCA) of ileocecal contents

Principal component analysis (PCA), a multivariate pattern recognition method, was employed to perform dimensionality reduction on rat serum positive (POS) and negative (NEG) ion mode data derived from different concentrations of DOC and its combination with antihypertensive chemical drugs (Supplementary Figure 2). Supplementary Figures 2A,B present the serum sample data from rats treated with varying concentrations of the DOC. The six samples at different concentrations are distributed across distinct regions, yielding a relatively ideal classification, indicating significant differences in chemical composition among these six samples. The samples exhibit a gradual transition trend along the PCA axes: the model group and the normal group are located in the positive direction of the PCA axis, whereas the treatment groups are positioned in the negative direction. Each group forms a distinct cluster, suggesting marked metabolic compositional differences between the treatment groups and both the normal and model groups. Moreover, clear distinctions are also observed among the various treatment groups. Supplementary Figures 2C,D display the POS-mode serum sample data from rats treated with the DOC in combination with antihypertensive chemical drugs. The samples again show a progressive distribution along the PCA axes. The two groups receiving only chemical drugs, as well as the combined traditional Chinese and Western medicine group, are clustered in the positive direction of the PCA axis. In contrast, the high-dose group, the irbesartan group, and the normal group are distributed in the negative direction. Each group forms a well-defined cluster, with highly evident intergroup separation, indicating significant differences in metabolic profiles among the groups and suggesting pronounced variations in therapeutic efficacy.

Supplementary Figure 3 presents the PCA of samples derived from various concentrations of DOC and its combination with antihypertensive chemical drugs. In Supplementary Figures 3A,B display the analytical data of samples from different concentrations of the DOC. The six samples at varying concentrations are distributed across distinct regions, exhibiting a favorable classification outcome, which indicates significant differences in chemical composition among these six samples. The samples show a gradual distribution pattern along the PCA axes and cluster into separate groups, suggesting notable differences in metabolic profiles among the high-, medium-, and low-concentration groups compared to the model group and the Irbesartan group. Moreover, the clear clustering of the high-, medium-, and low-concentration groups suggests a dose-effect relationship among them. C and D in Supplementary Figure 3 present the analytical data of samples from the combination of DOC with antihypertensive chemical drugs. The samples again exhibit a stepwise distribution trend along the PCA axes and form distinct clusters. Notably, the combined traditional Chinese and Western medicine group clusters closely with the high-concentration group, while the two Western medicine groups (Irbesartan group and normal control group) are relatively close to each other. This indicates that the metabolic profiles of the combined treatment group and the high-concentration group differ significantly from those of the Western medicine groups. These findings suggest that the traditional Chinese medicine group and the combined treatment group may modulate metabolic components in hypertensive rats through mechanisms distinct from those of the Western medicine groups. Furthermore, the separate clustering of the traditional Chinese medicine group and the combined treatment group indicates a clear metabolic distinction between these two groups as well.

### 3.4 Orthogonal partial least squares discriminant analysis (OPLS-DA) of serum and ileocecal contents in SHRs

To further investigate the differences in chemical constituents in rat serum and ileocecal contents following various administration methods, OPLS-DA models were constructed by comparing samples of serum and ileocecal contents from rats across different treatment groups, including various concentrations of DOC, combined traditional Chinese and Western medicine, and the model group. Pairwise comparisons between each intervention group and the model group were performed to identify differential metabolites. In the S-plot, compounds located farther from the origin and distributed toward the two extremes contribute more significantly to distinguishing between the groups. In the VIP plot, a higher VIP value indicates a greater contribution of individual chemical markers to the explained variance and a stronger association with intergroup differences. Differential compounds were selected based on a VIP threshold >1, in conjunction with analysis of the S-plot.

OPLS-DA analysis results and S-plots of serum and ileocecal contents POS and NEG samples from each rat group are shown in Supplementary Figures 4–11. The models were validated using cross-validation, and differential metabolites were identified based on a VIP value >1 in combination with the S-plots (Supplementary Tables 2–17).

### 3.5 Screening of blood pressure-related metabolites and metabolic pathways

**FIGURE 1:**
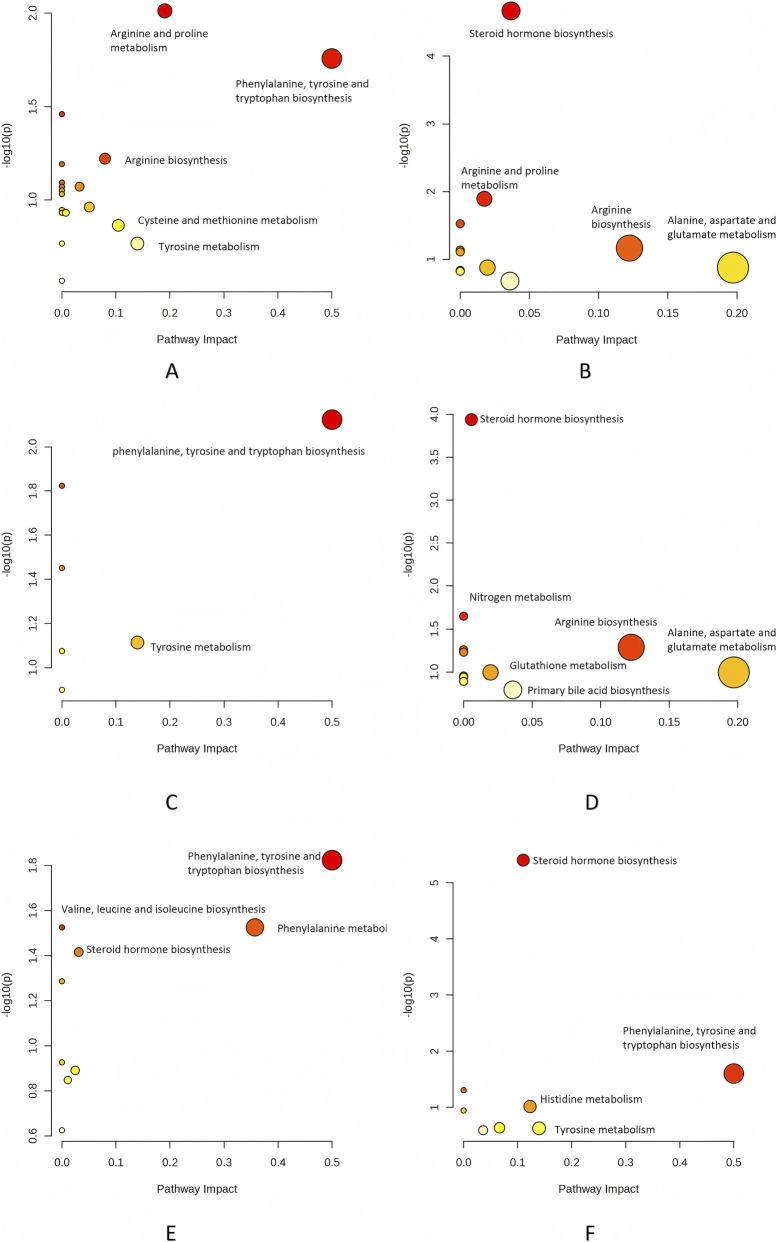
Scoring plot of differentially expressed metabolites and associated metabolic pathways in serum and ileocecal region of SHRs. Note: **(A,C,E)**: Score plots of serum common differentially abundant metabolites (**A**: MF vs. HF; **C**: HF vs. FIAC; **E**: metabolites increased in the FIAC group relative to the HF group). **(B,D,F)** Score plots of gut common differentially abundant metabolites (**B**: MF vs. HF; **D**: HF vs. FIAC; **F**: metabolites increased in the FIAC group relative to the HF group). In each plot, each circle represents a metabolic pathway. Circle color reflects the significance level (P-value). Circle size denotes the relative impact or enrichment magnitude of the pathway.

Based on the pharmacodynamic experimental results in SHRs, we observed that the antihypertensive efficacy of low-dose DOC was significantly lower than that of the medium- and high-dose groups. Moreover, blood pressure exhibited greater diurnal fluctuation in SHRs following intervention with the low-dose formulation, indicating an unstable antihypertensive effect. In contrast, the medium- and high-dose formulations demonstrated comparable and more stable therapeutic outcomes. Therefore, subsequent screening of metabolites from the MF, HF, and FIAC groups is more meaningful for elucidating the antihypertensive mechanisms of DOC monotherapy as well as combined traditional Chinese and Western medicine therapy. Integrating the serum and intestinal metabolomic data from SHRs, common differentially expressed metabolites were identified between the MF and HF groups, and between the HF and FIAC groups (Table 6; Table 7). Metabolic pathway analysis of these selected metabolites was performed using the MetaboAnalyst 6.0 database (Figure 1). The results indicated that, among the serum-related metabolic pathways shared by the MF and HF groups, phenylalanine, tyrosine, and tryptophan biosynthesis exhibited the greatest influence on antihypertensive efficacy (Figure 1A). In the intestinal metabolic pathways, alanine, aspartate, and glutamate metabolism, along with arginine biosynthesis, showed the most significant impact on blood pressure reduction (Figure 1B). Similarly, in the serum metabolic pathways shared by the HF and FIAC groups, phenylalanine, tyrosine, and tryptophan biosynthesis were the most influential pathways (Figure 1C), while in the intestinal pathways, alanine, aspartate, and glutamate metabolism, as well as arginine biosynthesis, exerted the greatest antihypertensive effects (Figure 1D). Notably, certain differentially expressed metabolites were uniquely present in the FIAC group. Screening these unique metabolites helps clarify the underlying mechanisms contributing to the enhanced antihypertensive efficacy when Western medication is combined with herbal treatment (Table 8; Figures 1E,F). Importantly, both in serum and intestinal metabolic pathways, phenylalanine, tyrosine, and tryptophan biosynthesis were found to have the greatest impact on the enhanced antihypertensive effect (Figure 1).

**TABLE 6:** Common differential metabolites in serum and ileocecal contents of SHRs in MF and HF groups.

​	Metabolite name	Metabolic pathway
Serum metabolites	L-Tyrosine	Phenylalanine metabolism
		Phenylalanine, tyrosine and tryptophan biosynthesis
		Ubiquinone and other terpenoid-quinone biosynthesis
		Tyrosine metabolism
	Spermidine	Arginine and proline metabolism
		Beta-alanine metabolism
		Glutathione metabolism
	L-Methionine	Cysteine and methionine metabolism
		One Carbon pool by folate
	Urate	Purine metabolism
	L-Arginine	Arginine and proline metabolism
		Arginine biosynthesis
	D-Mannose	Fructose and mannose metabolism
		Galactose metabolism
		Amino sugar and nucleotide sugar metabolism
	Succinate	Alanine, aspartate, and glutamate metabolism
		Propionate metabolism
		Citric acid cycle
		Butyrate metabolism
Gut metabolites	Pregnenolone	Steroid hormone biosynthesis
	Urocortisol	Steroid hormone biosynthesis
	11beta,17alpha,21-Trihydroxypregnenolone	Steroid hormone biosynthesis
	Tetrahydrocorticosterone	Steroid hormone biosynthesis
	Androstenediol	Steroid hormone biosynthesis
	3alpha,7alpha,12alpha,26-Tetrahydroxy-5beta-Cholestane	Primary Bile acid biosynthesis
	L-Proline	Arginine and proline metabolism
	L-Glutamate	Arginine and proline metabolism
		Nitrogen metabolism
		Arginine biosynthesis
		Propionate metabolism
		Histidine metabolism
		Glutathione metabolism
		Alanine, aspartate, and glutamate metabolism
		Porphyrin metabolism

**TABLE 7:** Common differential metabolites in serum and ileocecal contents of SHRs in HF and FIAC groups.

​	Metabolite name	Metabolic pathway
Serum metabolites	Urate	Purine metabolism
	Chenodeoxycholate	Primary bile acid biosynthesis
	L-Tyrosine	Phenylalanine, tyrosine and tryptophan biosynthesis
		Phenylalanine metabolism
		Ubiquinone and other terpenoid-quinone biosynthesis
		Tyrosine metabolism
Gut metabolites	Urocortisol	Steroid hormone biosynthesis
	11beta,17alpha,21-Trihydroxypregnenolone	Steroid hormone biosynthesis
	Androstenediol	Steroid hormone biosynthesis
	3alpha,7alpha,12alpha,26-Tetrahydroxy-5beta-Cholestane	Primary Bile acid biosynthesis
	Tetrahydrocorticosterone	Steroid hormone biosynthesis
	L-Glutamate	Nitrogen metabolism
		Arginine biosynthesis
		Butyrate metabolism
		Histidine metabolism
		Glutathione metabolism
		Alanine, aspartate, and glutamate metabolism
		Porphyrin metabolism
		Glyoxylate and Dicarboxylate metabolism
		Arginine and proline metabolism

**TABLE 8:** Differential metabolites increased in the FIAC group compared to the HF group.

​	Metabolite name	Metabolic pathway
Serum metabolites	Pregnenolone	Steroid hormone biosynthesis
	Creatine	Glycine, serine and threonine metabolism
		Arginine and proline metabolism
	Urea	Arginine biosynthesis
		Purine metabolism
	L-Phenylalanine	Phenylalanine, tyrosine and tryptophan biosynthesis
		Phenylalanine metabolism
	11-Dehydrocorticosterone	Steroid hormone biosynthesis
	4-Methyl-2-oxopentanoate	Valine, leucine and isoleucine biosynthesis
		Valine, leucine, and Isoleucine Degradation
Gut metabolites	3alpha,7alpha-Dihydroxy-5beta-cholestanate	Primary bile acid biosynthesis
	L-Tyrosine	Phenylalanine, tyrosine and tryptophan biosynthesis
		Phenylalanine metabolism
		Ubiquinone and other Terpenoid-Quinone biosynthesis
		Tyrosine metabolism
	Corticosterone	Steroid hormone biosynthesis
	Urocanate	Histidine metabolism
	17alpha-Hydroxypregnenolone	Steroid hormone biosynthesis
	17alpha,21-Dihydroxypregnenolone	Steroid hormone biosynthesis
	17alpha,21-Dihydroxy-5beta- pregnane-3,11,20-trione	Steroid hormone biosynthesis
	7alpha-Hydroxydehydroepiandrosterone	Steroid hormone biosynthesis

### 3.6 Effects of DOC and combined use of traditional Chinese and Western medicines on the intestinal microbial community structure in SHRs

We employed alpha and beta diversity analyses to determine whether the DOC, either alone or in combination with Western antihypertensive drugs, influenced the community structure and homeostasis of gut microbiota in SHRs. The Chao1, Richness, Shannon, and Simpson indices were selected to assess microbial species diversity. As shown in Supplementary Figures 12A–D, compared with the model group, the medium-dose group exhibited increased Chao1 and Richness indices, suggesting enhanced species richness and intergroup differentiation. Moreover, the Simpson index decreased while the Shannon index increased in the low-, medium-, and high-dose groups relative to the model group, indicating that intervention with the DOC led to an increase in gut microbial diversity in SHRs. Alpha diversity analysis of the combined treatment with the DOC and Western antihypertensive drugs (Supplementary Figures 12E–H) revealed that, compared with the model group, the combination group showed decreased values in Chao1, Richness, Simpson, and Shannon indices, suggesting a reduction in microbial abundance, intergroup variation, and overall diversity following intervention. These findings indicate that both different concentrations of the DOC and its combination with Western antihypertensive drugs exert significant effects on the richness of gut microbial communities in SHRs. It is therefore speculated that the antihypertensive efficacy of the DOC and its combination with Western medications may be mediated, at least in part, through modulation of gut microbiota composition and diversity.

Beta diversity analysis was performed using non-metric multidimensional scaling (NMDS) (Supplementary Figure 13). NMDS is a multivariate statistical method that reduces the complexity of high-dimensional data into a lower-dimensional space for visualization, analysis, and classification, while preserving the original dissimilarities among objects (samples or variables). Supplementary Figure 13A reveals distinct differences in microbial community composition between the model group and the treatment groups. Supplementary Figure 13B further demonstrates a clear separation between the combined traditional Chinese and Western medicine group and the compound formula group, providing additional evidence that the significant antihypertensive effects of DOC, particularly when used in combination with antihypertensive drugs, may be associated with alterations in gut microbiota structure.

**FIGURE 2:**
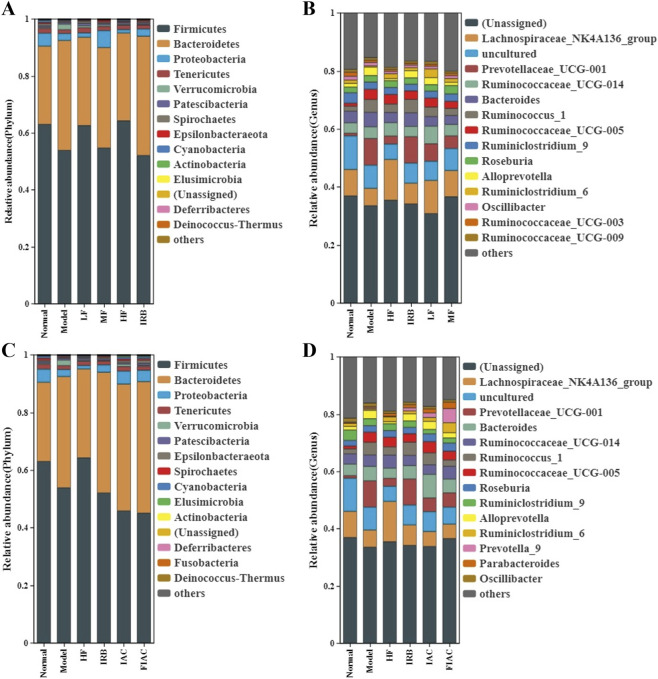
Histograms of relative abundances at the phylum and genus levels. Note: Histograms showing relative abundances at the phylum level **(A)** and genus level **(B)** under different DOC concentrations; histograms showing relative abundances at the phylum level **(C)** and genus level **(D)** under combined treatment with DOC and antihypertensive drugs.

**FIGURE 3:**
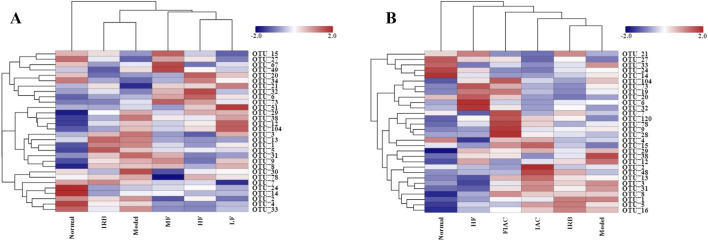
Species Abundance Clustering Heatmap. Note: **(A)** Clustering heatmap of species abundance across different DOC concentrations; **(B)** Clustering heatmap of species abundance under combined treatment with DOC and antihypertensive drugs. Color intensity reflects the relative abundance of each species within the respective samples: darker shades indicate higher relative abundance. The x-axis represents distinct intervention groups, and the y-axis represents individual species.

By comparing the relative abundance (RA) of microbial taxa at the phylum and genus levels across treatment groups, the impact of different concentrations of DOC alone, as well as combined traditional Chinese and Western medicine, on the gut microbiota community structure in SHRs was further investigated. Figures 2A,C show that, at the phylum level, the abundances of Firmicutes, Bacteroidetes, Proteobacteria, Tenericutes, and Verrucomicrobia were altered to varying degrees across groups, with the most notable changes observed in Firmicutes and Bacteroidetes. Following low-, medium-, and high-dose DOC interventions, the RA of Firmicutes in SHRs increased by 8.73%, 0.84%, and 10.40%, respectively, compared to the model group, while the RA of Bacteroidetes decreased by 7.60%, 3.31%, and 7.67%, respectively. In contrast, in the combined Chinese-Western medicine group, the RA of Firmicutes decreased by 8.80%, whereas that of Bacteroidetes increased by 7.05%. Figures 2B,D indicate that, at the genus level, DOC primarily modulated the abundances of Lachnospiraceae_NK4A136_group and Prevotellaceae_UCG-001 in the gut microbiota of SHRs. Specifically, the RA of Lachnospiraceae_NK4A136_group increased by 5.46%, 3.05%, and 8.06% following low-, medium-, and high-dose DOC treatments, respectively, compared to the model group, while Prevotellaceae_UCG-001 decreased by 3.15%, 4.83%, and 6.39%. In the combined therapy group, the most pronounced changes were observed in Prevotellaceae_UCG-001 and Prevotella_9: the RA of Prevotellaceae_UCG-001 decreased by 4.18%, while that of Prevotella_9 increased by 4.49% relative to the model group. A heatmap based on species abundance clustering was used to visualize the community composition and abundance profiles at the OTU level across all groups (Figure 3).

Linear discriminant analysis Effect Size (LEfSe), also known as LDA Effect Size analysis, is a computational method designed to identify and interpret biomarkers in high-dimensional datasets. It enables comparative analyses between two or more groups, as well as among subgroups within a given group, thereby facilitating the detection of microbial taxa that exhibit significant abundance differences across groups. In this study, LEfSe analysis was applied to compare the relative abundances of gut microbiota among the low-, medium-, and high-dose groups, the combined traditional Chinese and Western medicine group, and the model group in rats. This approach aimed to identify potential differential microbial taxa associated with varying concentrations of DOC intervention in hypertension, as well as those microbial signatures linked to enhanced antihypertensive effects following combined pharmacotherapy (Supplementary Figure 14; Tables 9–12).

**TABLE 9:** Abundance of gut microbiota in rats following intervention with different concentrations of DOC: LEfSe analysis of differential microbial taxa between LF and Model groups.

No.	Differential microbial taxa names	Phylum	-log10(Abundance)	LDA	p
1	*Christensenellaceae R 7 group uncultured Bacterium*	*Firmicutes*	3.715243109	3.089239	0.037373
2	*Lachnospiraceae NC2004 group uncultured Bacterium*	*Firmicutes*	1.862411901	3.038375	0.010406
3	*Lachnospiraceae NC2004 group*	*Firmicutes*	1.862411901	3.038375	0.010406
4	*Lachnospiraceae NK4A136 group*	*Firmicutes*	4.961779425	4.363158	0.024975
5	*Parvibacter uncultured bacterium*	*Bacteroidetes*	1.509148819	3.251277	0.04632
6	*Parvibacter sp.*	*Bacteroidetes*	1.509148819	3.241609	0.04632
7	*Ruminiclostridium 6 uncultured bacterium*	*Firmicutes*	4.379784402	4.000145	0.006485
8	*Ruminiclostridium 6 sp.*	*Firmicutes*	4.379866944	4.000212	0.006485

**TABLE 10:** Abundance of gut microbiota in rats following intervention with different concentrations of DOC: LEfSe analysis of differential microbial taxa between MF and Model groups.

No.	Differential microbial taxa names	Phylum	-log10(Abundance)	LDA	p
1	*Alistipes uncultured bacterium*	*Bacteroidetes*	3.713105471	3.042315	0.010406
2	*Alistipes sp.*	*Bacteroidetes*	3.735091209	3.061334	0.010406
3	*Desulfovibrio uncultured bacterium*	*Proteobacteria*	3.579886293	3.143707	0.016309
4	*Desulfovibrio sp.*	*Proteobacteria*	3.584290462	3.148081	0.016309
5	*Enterobacter sp.*	*Proteobacteria*	4.532630557	4.335001	0.010406
6	*Parabacteroides uncultured bacterium*	*Bacteroidetes*	3.930817003	3.511685	0.003948
7	*Parabacteroides sp.*	*Bacteroidetes*	4.034563298	3.517039	0.003948
8	*RB41 sp.*	*Proteobacteria*	0.994357706	3.015093	0.04951
9	*Ruminiclostridium 5 sp.*	*Firmicutes*	3.551197434	3.043903	0.010406

**TABLE 11:** Abundance of gut microbiota in rats following intervention with different concentrations of DOC: LEfSe analysis of differential microbial taxa between HF and Model groups.

No.	Differential microbial taxa names	Phylum	-log10(Abundance)	LDA	p
1	*Anaerostipes uncultured bacterium*	*Firmicutes*	4.189168	3.774843	0.024975
2	*Anaerostipes* sp.	*Firmicutes*	4.189259	3.774953	0.024975
3	*Lachnospiraceae NK4A136 group uncultured bacterium*	*Firmicutes*	4.947237	4.38486	0.010406
4	*Lachnospiraceae NK4A136 group*	*Firmicutes*	5.080122	4.524935	0.006485
5	*Marvinbryantia uncultured bacterium*	*Firmicutes*	3.585127	3.156163	0.024975
6	*Marvinbryantia sp.*	*Firmicutes*	3.611822	3.183998	0.024975
7	*Ruminiclostridium 6 uncultured bacterium*	*Firmicutes*	4.164594	3.747446	0.006485
8	*Ruminiclostridium 6 sp.*	*Firmicutes*	4.164594	3.747446	0.006485
9	*Turicibacter uncultured bacterium*	*Firmicutes*	3.363091	3.005363	0.016309
10	*Turicibacter sp.*	*Firmicutes*	3.363091	3.005363	0.016309

**TABLE 12:** Abundance of gut microbiota in rats following intervention with different concentrations of DOC: LEfSe analysis of differential microbial taxa between FIAC and Model groups.

NO.	Differential microbial taxa names	Phylum	-log10(Abundance)	LDA	p
1	*Elusimicrobium uncultured bacterium*	*Elusimicrobia*	3.536861041	3.157533	0.010406
2	*Elusimicrobium* sp.	*Elusimicrobia*	3.536861041	3.157533	0.010406
3	*Marvinbryantia uncultured bacterium*	*Firmicutes*	3.629157522	3.191761	0.037373
4	*Marvinbryantia sp.*	*Firmicutes*	3.663710253	3.231344	0.037373
5	*Parabacteroides uncultured bacterium*	*Bacteroidetes*	4.153012835	3.783736	0.003948
6	*Parabacteroides sp.*	*Bacteroidetes*	4.324066991	3.926724	0.003948
7	*Parasutterella gut metagenome*	*Bacteroidetes*	3.73565023	3.260968	0.010406
8	*Parasutterella sp.*	*Bacteroidetes*	3.840774663	3.383615	0.006485
9	*Prevotella 1 uncultured bacterium*	*Bacteroidetes*	3.886438869	3.482262	0.037373
10	*Prevotella 1 sp.*	*Bacteroidetes*	3.886438869	3.482262	0.037373
11	*Prevotella 9 sp.*	*Bacteroidetes*	4.635866223	4.269387	0.037373

## 4 Discussion

Despite the increasingly mature development of antihypertensive Western medications, the global blood pressure control rate among hypertensive patients remains suboptimal. TCM, as an adjunctive or alternative antihypertensive approach, offers distinctive advantages—stemming from its multi-component, multi-target holistic regulatory effects. Not only can TCM help lower blood pressure, but it also effectively alleviates associated symptoms such as dizziness and headache. Moreover, TCM excels in comprehensive physiological regulation and enhancement of patients’ overall quality of life. *Dendrobium officinale*, a traditional Chinese medicine with dual medicinal and dietary applications (i.e., approved for both therapeutic use and incorporation into daily diets), exhibits a favorable safety profile. However, international research on its antihypertensive effects remains limited and insufficient. Building upon the prior work, this study further investigates the underlying blood pressure-lowering mechanisms of D. officinale—both as a monotherapy and in combination with conventional Western antihypertensive drugs (Wu et al., 2025), thereby aiming to address this critical knowledge gap in the field. A substantial body of research has elucidated the underlying pathogenic mechanisms of primary hypertension, primarily encompassing: (i) overactivation of the renin–angiotensin–aldosterone system (RAAS); (ii) structural and functional abnormalities of blood vessels; (iii) dysregulated immune and inflammatory responses; (iv) gut microbiota dysbiosis; and (v) disturbances in amino acid transport and metabolism (Patel et al., 2017; Bleakley et al., 2015; Pacella et al., 2024; Kuwabara et al., 2025). This study demonstrates that DOC exerts a significant and sustained antihypertensive effect in SHRs, with the optimal dose identified at 2.8 g/kg. Furthermore, the antihypertensive efficacy of DOC is enhanced when co-administered with the Western antihypertensive agents irbesartan and amlodipine besylate. Metabolomic analyses revealed that, compared with the model control group, both the medium- and high-dose DOC groups—as well as the DOC combined with Western medicine group—exhibited significant alterations in serum metabolite profiles, prominently featuring multiple amino acids. Similarly, differential metabolites detected in ileocecal contents also included diverse amino acids. These findings collectively suggest that DOC may exert its antihypertensive effects, at least in part, by modulating systemic amino acid composition and associated metabolic pathways. Notably, upon combination with Western antihypertensive drugs, the differential metabolite profiles in both serum and ileocecal contents continued to feature numerous amino acids, while additionally encompassing several metabolites involved in steroid hormone metabolism. RNA-based gut microbiota analysis further indicated that Bacteroidetes and Firmicutes were the predominant phyla exhibiting significant abundance shifts following DOC monotherapy or DOC–Western medicine combination intervention. Such microbiota remodeling likely contributes directly to the observed alterations in gut and systemic metabolite profiles, thereby underpinning the antihypertensive mechanism of DOC.

Studies have shown that medicinal *Dendrobium officinale* is exceptionally rich in amino acids, with particularly high levels of glutamic acid, glycine, valine, and leucine. Notably, the amino acid content in D. officinale flowers exceeds that in the stems, and arginine and proline are the most abundant amino acids in the flowers (Yuan et al., 2019). In fact, human cells possess the capacity to synthesize several amino acids—termed non-essential amino acids—including alanine, arginine, proline, and tyrosine (Cunnane, 2003). Conversely, certain amino acids—such as methionine, phenylalanine, and leucine—cannot be synthesized endogenously and must therefore be obtained through dietary intake; these are classified as essential amino acids. Integrating our experimental findings, we observed that multiple amino acids—including tyrosine, proline, methionine, glutamic acid, arginine, and phenylalanine—as well as their associated metabolic pathways, play critical roles in the antihypertensive effects of DOC, both as monotherapy and in combination with conventional Western medications. Importantly, this set encompasses both essential and non-essential amino acids. Accordingly, we hypothesize that DOC exerts its blood pressure-lowering effects not only by modulating serum levels of specific non-essential amino acids and thereby altering the activity of related metabolic pathways, but also by supplementing essential amino acids that promote antihypertensive action.

Tyrosine and its precursor phenylalanine are aromatic amino acids that play critical roles in neurotransmitter synthesis as well as in the structural integrity and functional properties of proteins. Their metabolic pathways also contribute significantly to blood pressure regulation. Hao et al. reported a marked reduction in plasma phenylalanine levels among hypertensive patients (Hao et al., 2016). Phenylalanine further modulates the biosynthesis of tetrahydrobiopterin (BH4) (Mitchell et al., 2004). In a study using Dahl salt-sensitive (SS) rats fed a high-salt diet, supplementation with L-phenylalanine restored depleted BH4 and nitrite levels and ameliorated impaired endothelium-dependent vasorelaxation to acetylcholine. Moreover, in the kidneys of hypertensive SS rats treated with L-phenylalanine, mRNA expression of both BH4 and GTP cyclohydrolase I (GCH1)—the rate-limiting enzyme in BH4 biosynthesis—was upregulated, while superoxide production was significantly reduced. Collectively, these findings suggest that the antihypertensive effect of L-phenylalanine may be mediated through enhanced BH4 biosynthesis and concomitant attenuation of superoxide generation by uncoupled nitric oxide synthase, thereby alleviating salt-induced hypertension in SS rats (Wang et al., 2021).

Proline is currently widely recognized to participate in multiple physiological processes, including apoptosis, redox homeostasis, and cellular proliferation and differentiation (Patriarca et al., 2021); it is also considered essential for vascular remodeling (Li et al., 2001). Moreover, proline functions as an antioxidant: through the arginine–proline metabolic pathway, it upregulates antioxidant enzymes, scavenges reactive oxygen species (ROS), and thereby enhances cellular antioxidant capacity (El-Bassossy et al., 2012). In hypertensive rat models, supplementation with an appropriate dose of proline has been demonstrated to significantly increase nitric oxide (NO) bioavailability, effectively counteracting angiotensin II-induced hypertension (Le et al., 2019). Notably, sustained elevations in proline and tyrosine levels have been observed in hypertensive populations (Mahbub et al., 2019). Moreover, tyrosine—serving as a key precursor for catecholamine synthesis—is an essential component of the sympathetic nervous system and may exacerbate hypertension through its role in mediating vasoconstriction (Joyner et al., 2010). In a human metabolomic study investigating metabolic risk factors, a negative correlation was identified between the glutamine-to-glutamate ratio and blood pressure, lending indirect support to the notion that glutamate may exert a pro-hypertensive effect (Cheng et al., 2012). Integrating these findings with our own results, we propose the following interpretations: First, although this study identified several differentially expressed amino acids, not all amino acids exhibit antihypertensive activity. This is attributable to the chemical complexity and diversity of bioactive constituents present in traditional Chinese medicine (TCM) compound formulas, which collectively confer multifaceted pharmacological actions. Second, amino acids—and the metabolic pathways in which they participate—form an intricate, interdependent network; homeostatic regulation of blood pressure relies on the functional equilibrium within this network. Disruption of such equilibrium represents one potential pathogenic mechanism underlying hypertension. DOC appears to modulate the distribution profiles of amino acids, thereby facilitating the restoration of this equilibrium and contributing to its antihypertensive effects.

The gut microbiota constitutes a vast and highly diverse microbial community that dynamically responds to host physiological changes while simultaneously modulating host metabolic processes. A delicate equilibrium in gut microbial composition is essential for maintaining intestinal immune homeostasis and systemic physiological stability; disruption of this balance has been implicated in a spectrum of metabolic disorders (Tang et al., 2013; Tilg and Kaser, 2011). Among the dominant bacterial phyla in the human gut, Firmicutes and Bacteroidetes are particularly well characterized. Accumulating evidence indicates that the *Firmicutes*-to-*Bacteroidetes* ratio (F/B ratio) serves as a potential biomarker for certain pathological conditions (Sanz and Moya-Pérez, 2014). Besides, significant differences in the F/B ratio have been consistently observed—both in SHRs compared with normotensive control strains (e.g., Wistar rats) and in human cohorts with hypertension versus those without (Yang et al., 2015; Li et al., 2017; Tsiavos et al., 2024). Thus, the relative abundances of *Firmicutes* and *Bacteroidetes* hold substantial relevance for elucidating the pharmacological mechanisms underlying novel antihypertensive agents. One plausible mechanism by which gut microbiota may exert antihypertensive effects involves the release of bioactive metabolites—including amino acids—upon microbial cell death or lysis. These metabolites, even at low systemic concentrations following intestinal absorption, may contribute to blood pressure regulation. This hypothesis is supported by our experimental findings: SHRs treated with either DOC or a combination of traditional Chinese and Western medicine exhibited significantly elevated colonic glutamate levels—a key amino acid involved in butyrate metabolism. Butyrate, a major short-chain fatty acid (SCFA), has been robustly demonstrated to confer protective effects against hypertension (Muralitharan et al., 2025). Alternatively, DOC may directly supply exogenous SCFAs, thereby altering the luminal microenvironment and subsequently reshaping gut microbial community structure and amino acid profiles.

Changes in the relative abundances of the phyla *Firmicutes* and *Bacteroidetes* directly reflect shifts in the abundances of their constituent bacterial strains. Following intervention with varying concentrations of DOC, significant increases were observed in several *Firmicutes* taxa, including the *Christensenellaceae R 7 group uncultured Bacterium*, *Ruminiclostridium 5 sp.*, and *Anaerostipes uncultured bacterium*. In contrast, combined traditional Chinese and Western medicine intervention led to marked enrichment of multiple *Bacteroidetes* taxa, such as *Parabacteroides uncultured bacterium*, *Parabacteroides sp.*, and *Parasutterella gut metagenome*. Compared with women with hypertension, healthy individuals exhibited significantly higher abundance of *Ruminiclostridium*, a genus known to participate in diverse metabolic pathways—including lipid, amino acid, and carbohydrate metabolism (Louca et al., 2021), although the study population in this investigation exhibits certain limitations and the findings do not directly establish a causal role for *Ruminiclostridium* in blood pressure reduction, our results allow for a preliminary inference that DOC may elevate the abundance of *Ruminiclostridium* through multiple mechanisms—including provision of essential nutrients, modulation of the gut microenvironment, and suppression of competing pathogenic bacteria—thereby enabling its participation in metabolic pathways associated with blood pressure lowering. The beneficial effects of gut microbiota on blood pressure regulation have already been leveraged clinically in fecal microbiota transplantation (FMT). Notably, patients exhibiting significant reductions in blood pressure following FMT were found to harbor markedly increased abundances of *Parabacteroides merdae*, *Prevotella copri*, and *Bacteroides galacturonicus* in their gut microbiomes; these bacterial taxa are also significantly correlated with metabolites such as tyrosine, glutamine, aspartic acid, and phenylalanine (Fan et al., 2025).

We hypothesize that the aforementioned phenomena are not isolated but are associated with the gut microbiota and host metabolism. A plausible mechanistic explanation is that bioactive constituents in DOC—such as polysaccharides and alkaloids—directly or indirectly promote the proliferation of specific beneficial bacterial taxa, including short-chain fatty acid (SCFA)-producing genera. These microbiota alterations may exert dual effects: first, modulating intestinal amino acid metabolism and absorption; and second, releasing microbial metabolites—particularly SCFAs—into systemic circulation, thereby regulating hepatic and renal amino acid metabolic kinetics in the host. Consequently, serum levels of functionally active amino acids—such as those with vasodilatory or endothelium-protective properties—are elevated, collectively contributing to the antihypertensive effect.

Our study not only elucidates the enhanced antihypertensive mechanism underlying the combination therapy of DOC and Western antihypertensive drugs—namely, modulation of gut microbiota composition and host metabolic status—but also provides a scientific foundation for developing personalized antihypertensive regimens based on TCM compound formulas, demonstrating promising potential for clinical translation. However, we acknowledge certain limitations in our study. Isobolographic analysis represents a rigorous pharmacological approach that enables quantitative classification of drug interactions as enhanced, additive, or antagonistic. Nevertheless, this method was not employed in the present study, for the following reasons: First, isobolographic analysis typically relies on well-defined dose–response curves for drugs acting on a shared pharmacological target—a prerequisite that is difficult to fulfill for multi-component botanical mixtures such as DOC. Second, our experimental design focused on fixed-combination regimens of DOC and antihypertensive agents to investigate alterations in the gut microbiota and associated metabolic pathways; it was not optimized for the multiple-dose-ratio combinations required for isobologram construction. Third, the observed antihypertensive effects may arise from distinct yet complementary mechanisms: DOC appears to exert its effects via the gut microbiota–host metabolic axis, including modulation of short-chain fatty acid production and diverse amino acid metabolism pathways. In future studies, we plan to incorporate multiple dosing levels of both conventional antihypertensive drugs and DOC, and apply isobolographic analysis to characterize the dose–efficacy relationship—thereby identifying optimal combination doses and refining therapeutic strategies.

## 5 Conclusion

To our knowledge, this is one of the studies demonstrating that DOC intervention ameliorates hypertension in SHRs. Moreover, combining DOC with conventional antihypertensive drugs further enhances blood pressure-lowering efficacy. This enhanced effect may be attributable to DOC’s ability to specifically modulate serum amino acid profiles—including tyrosine, proline, and arginine—in SHRs and to reshape gut microbiota composition. Notably, both serum tyrosine and phenylalanine, as well as intestinal tyrosine, are integral components of the phenylalanine, tyrosine, and tryptophan biosynthesis and metabolism pathway. These findings provide novel mechanistic insights into the antihypertensive actions of traditional herbal medicine.

## Data Availability

The metabolomics data have been deposited to MetaboLights repository with the study identifier MTBLS14513. The 16S rRNA sequence files and associated metadata generated in this study have been securely deposited in the NCBI Sequence Read Archive (SRA) repository (BioProject accession: PRJNA1465535).
