# Supplementary material for: A mechanistic study on the enhanced antihypertensive effects of a Dendrobium officinale compound combined with Western antihypertensive drugs in spontaneously hypertensive rats based on metabolomics and gut microbiota analysis
Source: Front Cell Dev Biol. 2026 Jun 15;14:1806395. doi: 10.3389/fcell.2026.1806395 (PMC13315239; doi:10.3389/fcell.2026.1806395)
Supplement: Supplementary file 1 [file DataSheet1.doc]

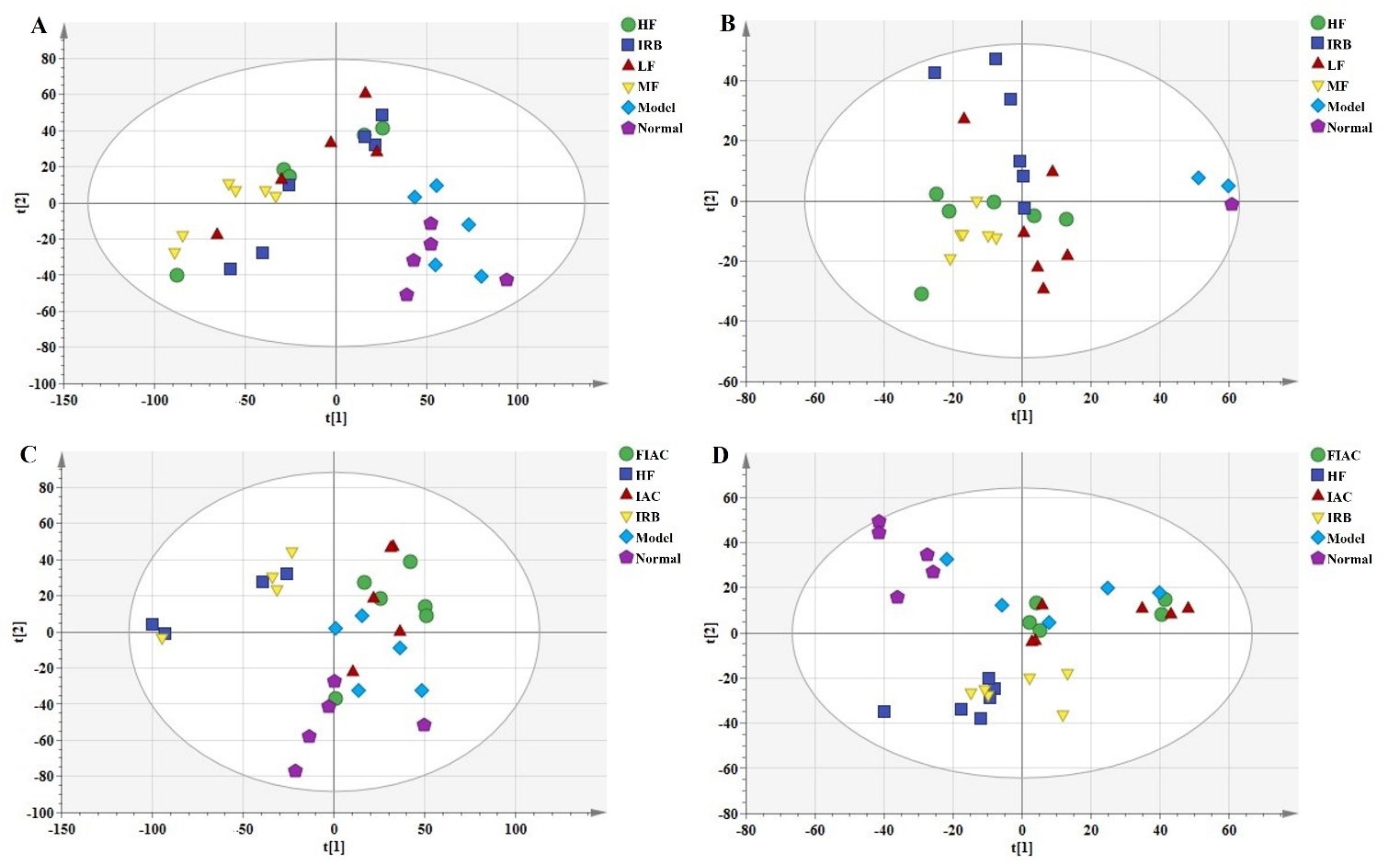


Supplementary Figure 1 Figure 1 Principal Component Analysis (PCA) plots of rat serum samples. (A and B: PCA results of serum samples from rats treated with different concentrations of DOC in positive mode (A) and negative mode (B); C and D: PCA results of serum samples from rats treated with the DOC in combination with antihypertensive drugs in positive mode (C) and negative mode (D). Normal: normal control group; Model: model group; LF: low-dose DOC group; MF: medium-dose DOC group; HF: high-dose DOC group; IRB: irbesartan group; IAC: two Western medicine group; FIAC: combined traditional Chinese and Western medicine group).


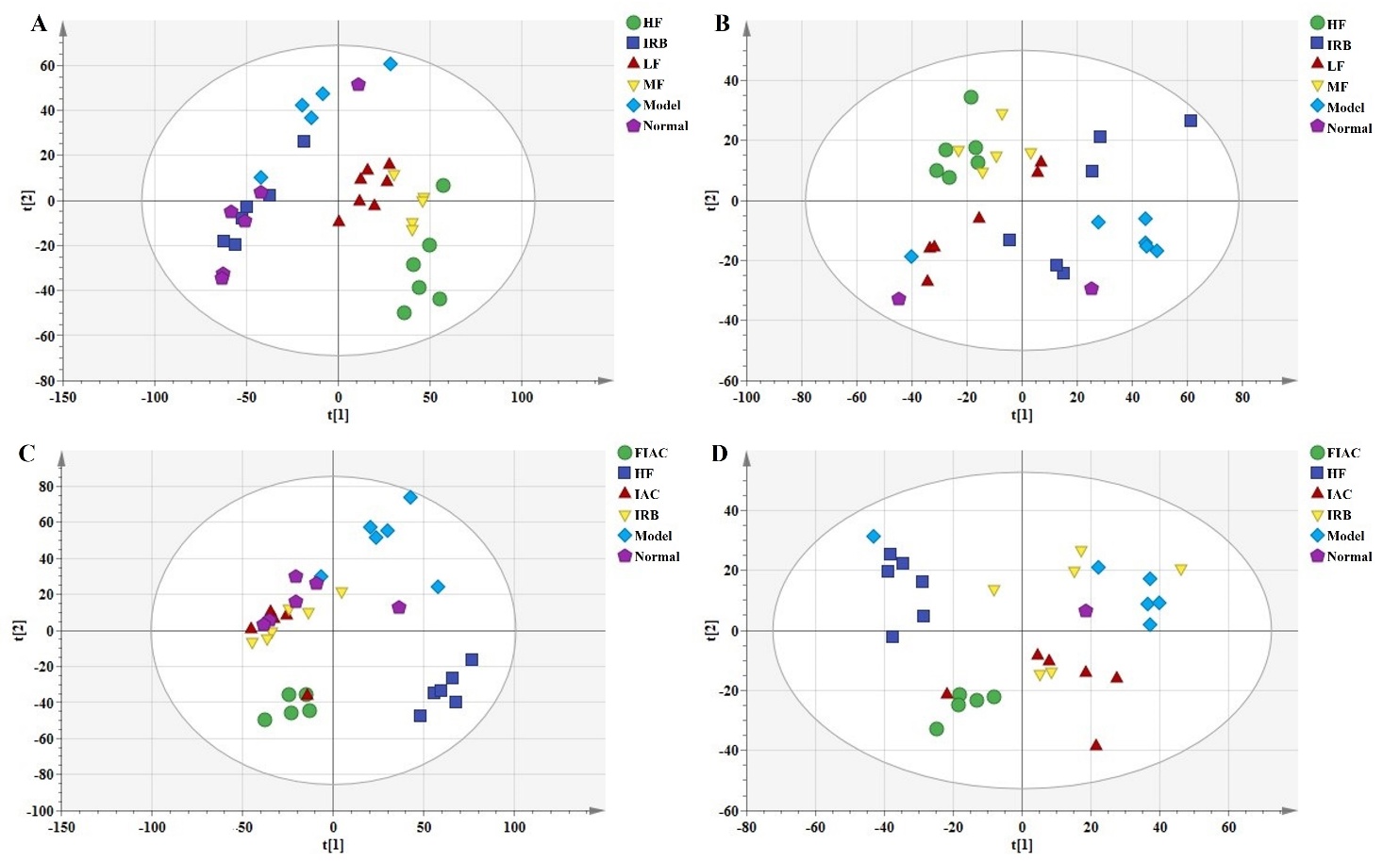


Supplementary Figure 2 Principal Component Analysis (PCA) plot of ileocecal content samples from rats. (A and B: PCA results of serum samples from rats treated with different concentrations of DOC in positive mode (A) and negative mode (B); C and D: PCA results of serum samples from rats treated with the DOC in combination with antihypertensive drugs in positive mode (C) and negative mode (D). Normal: normal control group; Model: model group; LF: low-dose DOC group; MF: medium-dose DOC group; HF: high-dose DOC group; IRB: irbesartan group; IAC: two Western medicine group; FIAC: combined traditional Chinese and Western medicine group).


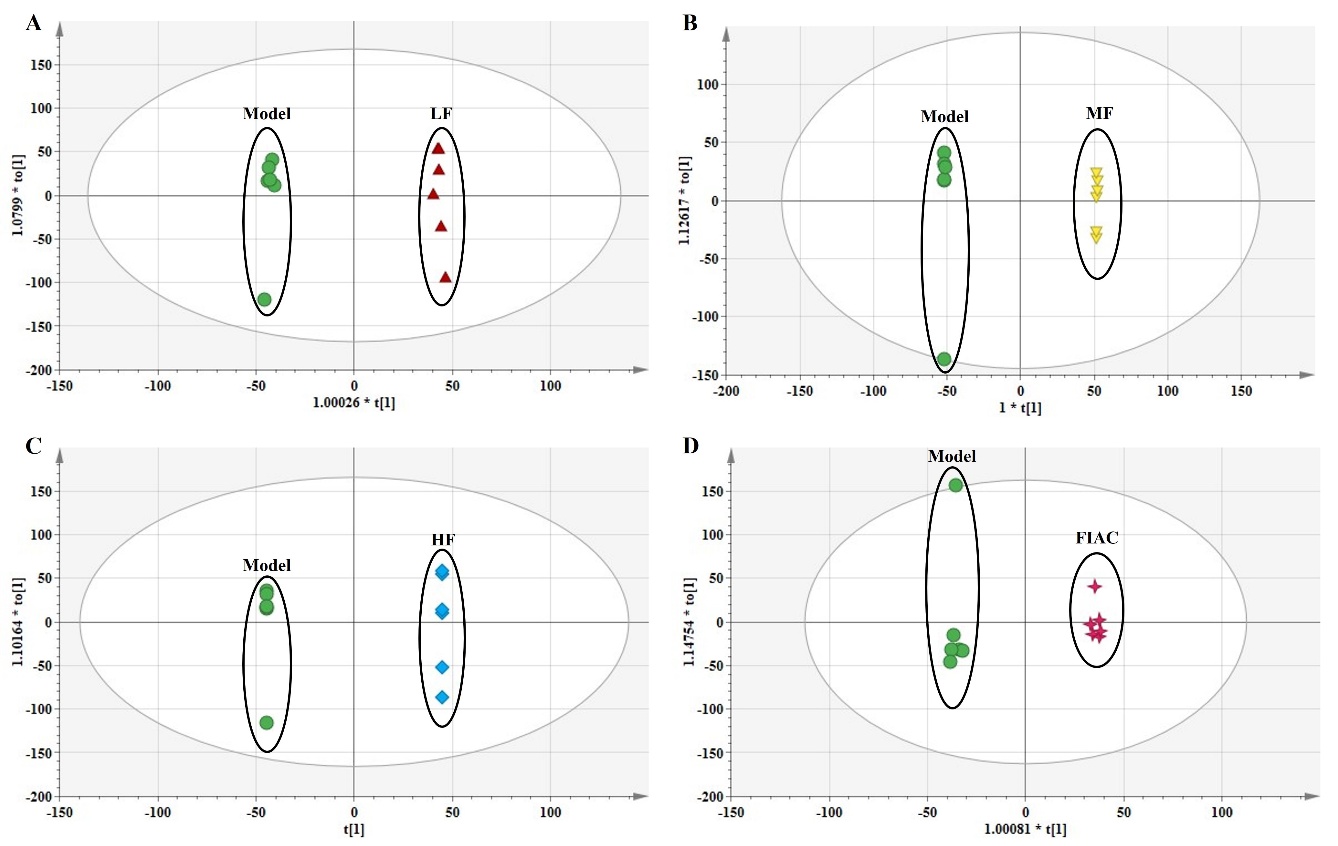


Supplementary Figure 3 OPLS-DA score plot of serum POS from rats treated with different intervention protocols

Note: A: LF vs Model; B: MF vs Model; C: HF vs Model; D: FIAC vs Model


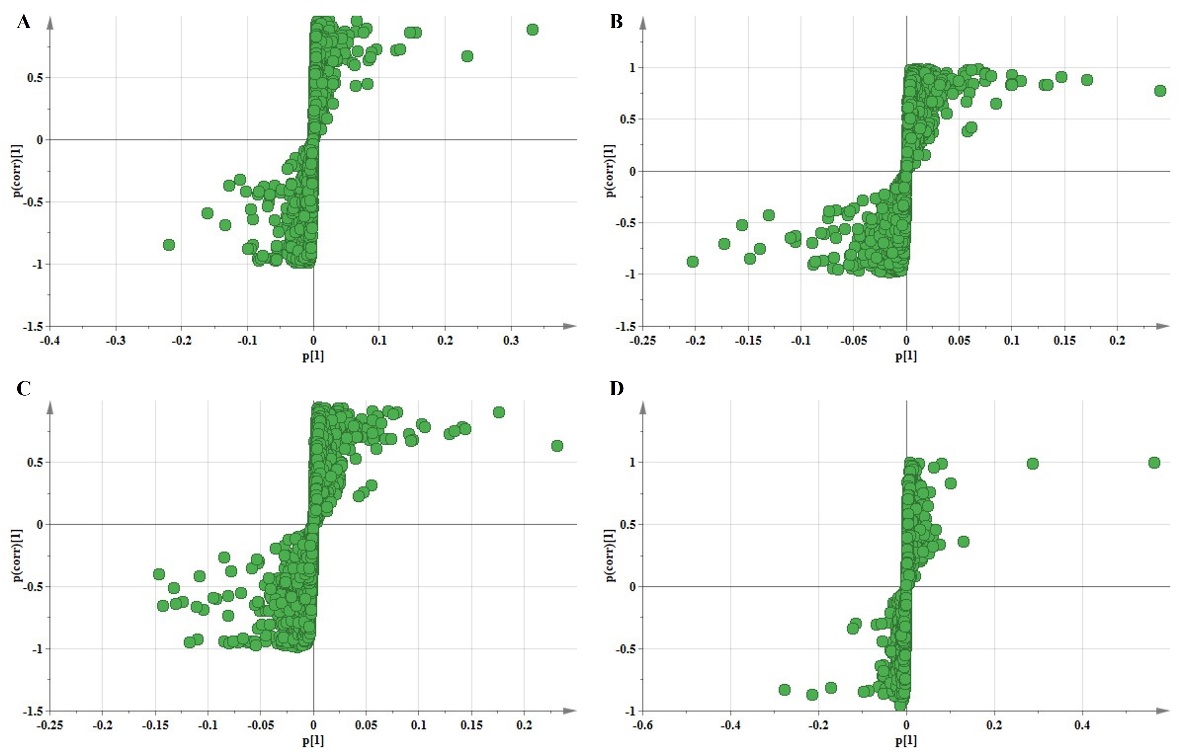


Supplementary Figure 4 Figure 4 S-plot score plot of serum POS from rats treated with different intervention protocols

Note: A: LF vs Model; B: MF vs Model; C: HF vs Model; D: FIAC vs Model


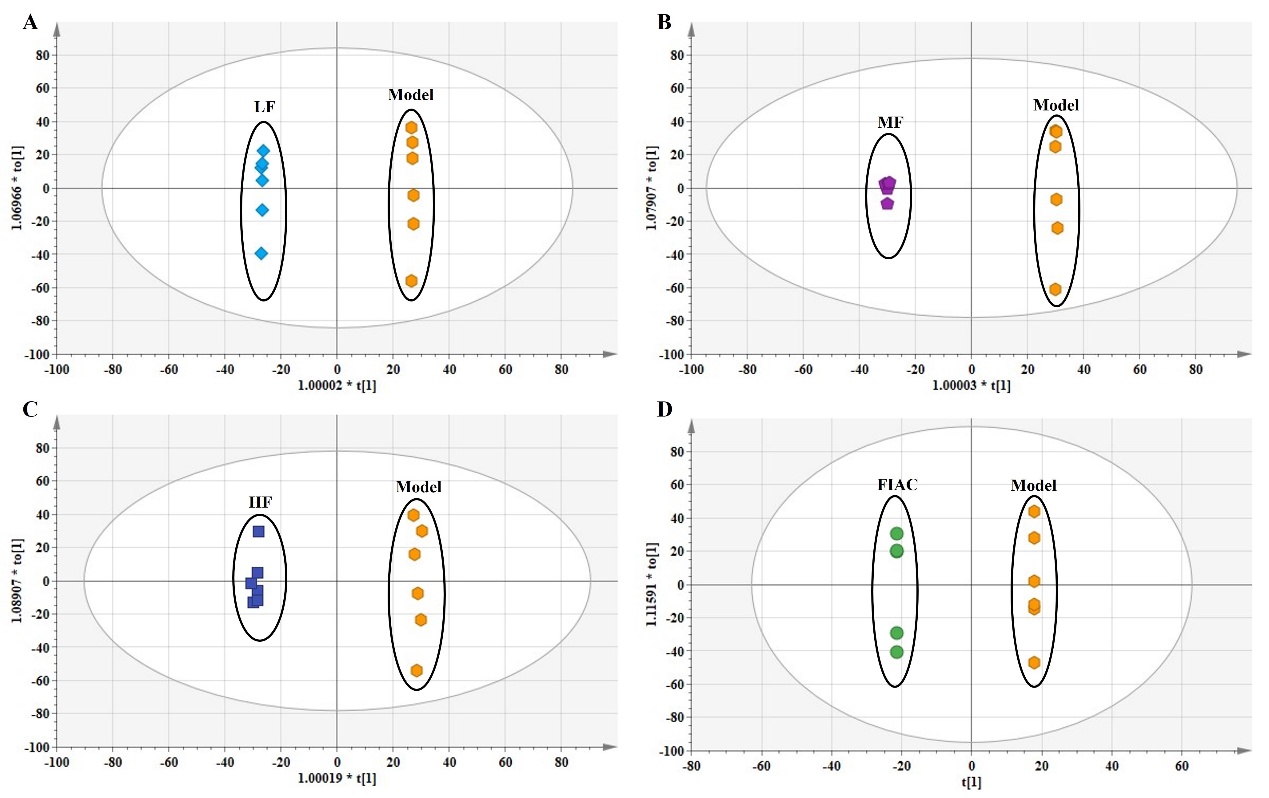


Supplementary Figure 5 OPLS-DA score plot of serum NEG from rats treated with different intervention protocols

Note: A: LF vs Model; B: MF vs Model; C: HF vs Model; D: FIAC vs Model


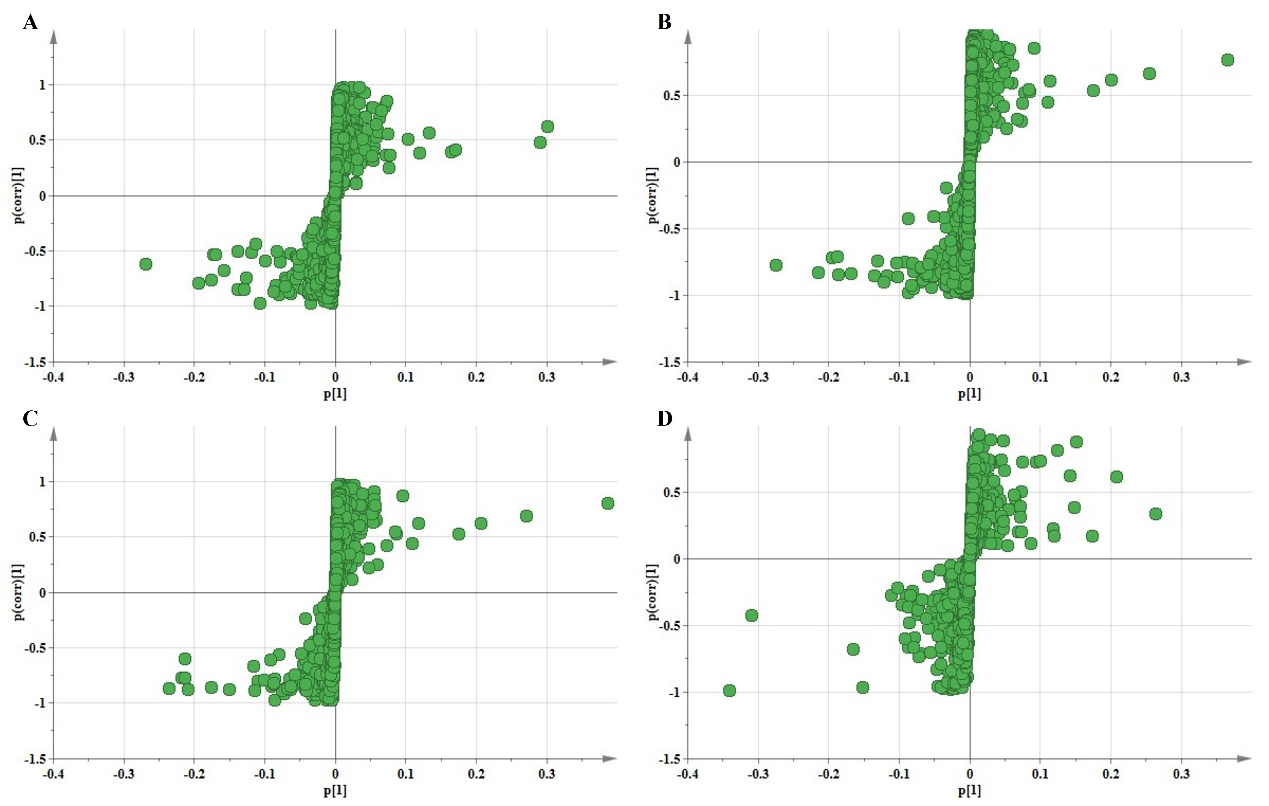


Supplementary Figure 6 S-plot score plot of serum NEG from rats treated with different intervention protocols

Note: A: LF vs Model; B: MF vs Model; C: HF vs Model; D: FIAC vs Model


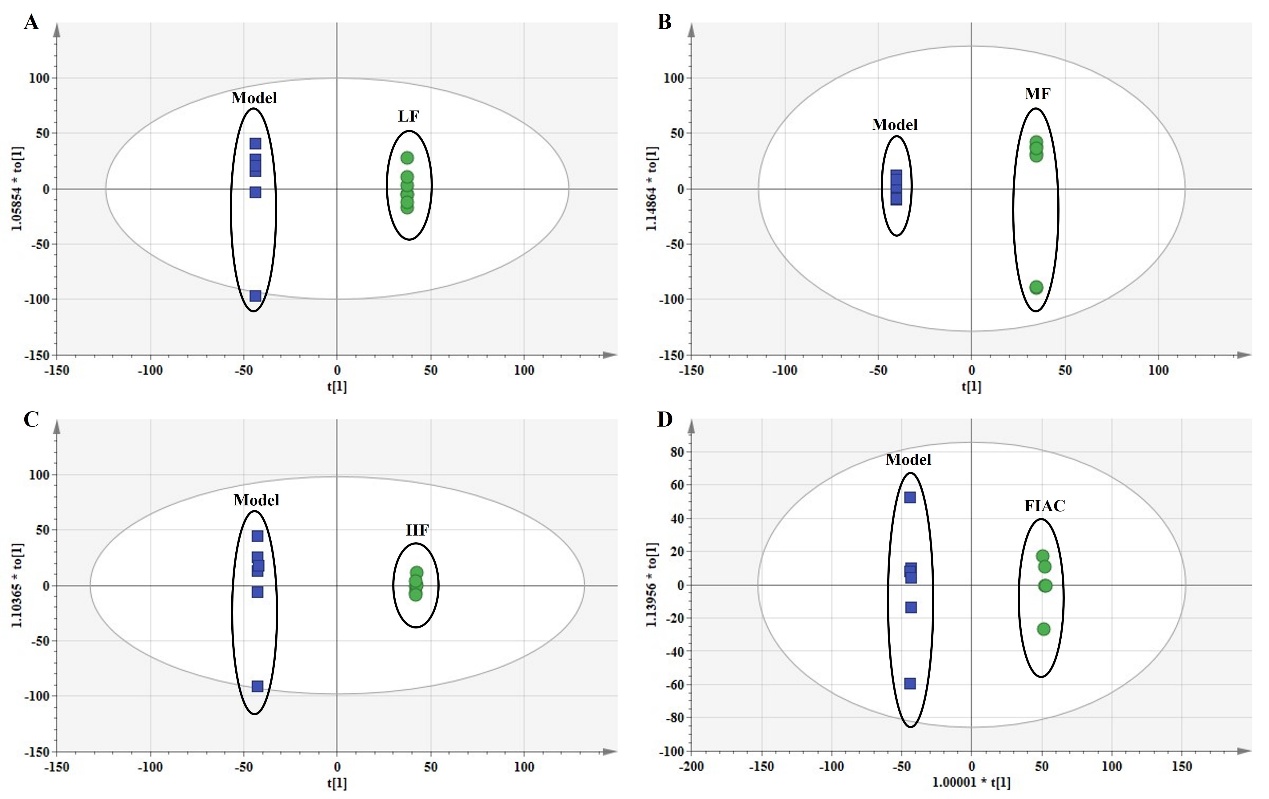


Supplementary Figure 7 OPLS-DA score plot of ileocecal contents POS from different samples in rats subjected to various intervention protocols

Note: A: LF vs Model; B: MF vs Model; C: HF vs Model; D: FIAC vs Model


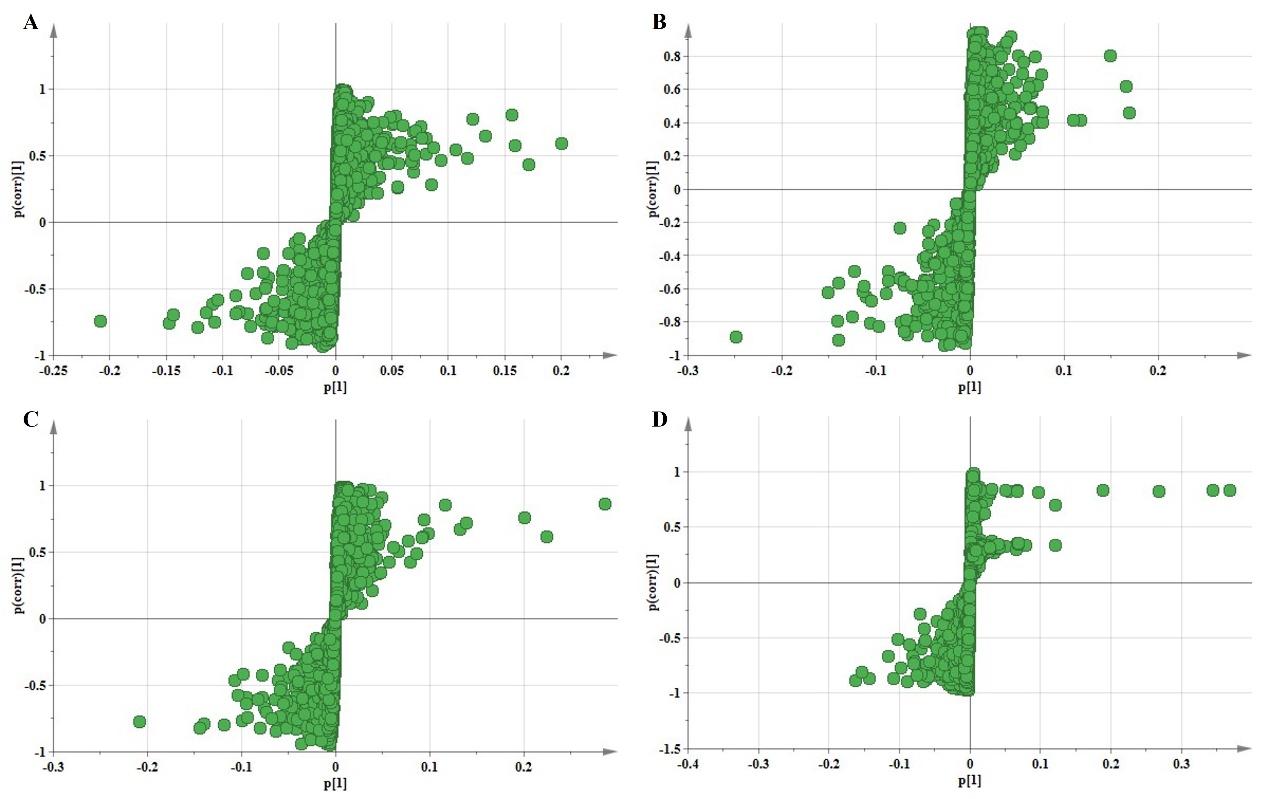


Supplementary Figure 8 S-plot score plot of ileocecal contents POS from different samples in rats subjected to various intervention protocols

Note: A: LF vs Model; B: MF vs Model; C: HF vs Model; D: FIAC vs Model


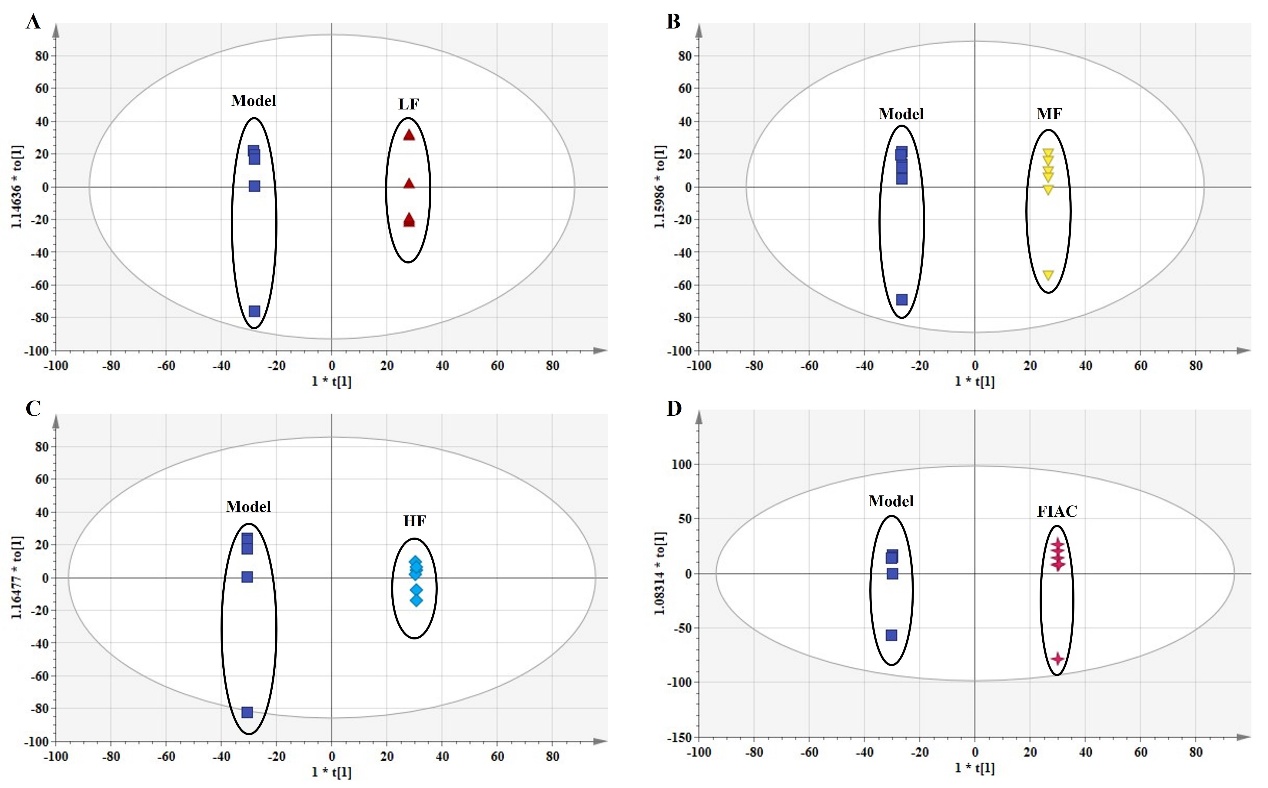


Supplementary Figure 9 OPLS-DA score plot of ileocecal contents NEG from different samples in rats subjected to various intervention protocols

Note: A: LF vs Model; B: MF vs Model; C: HF vs Model; D: FIAC vs Model


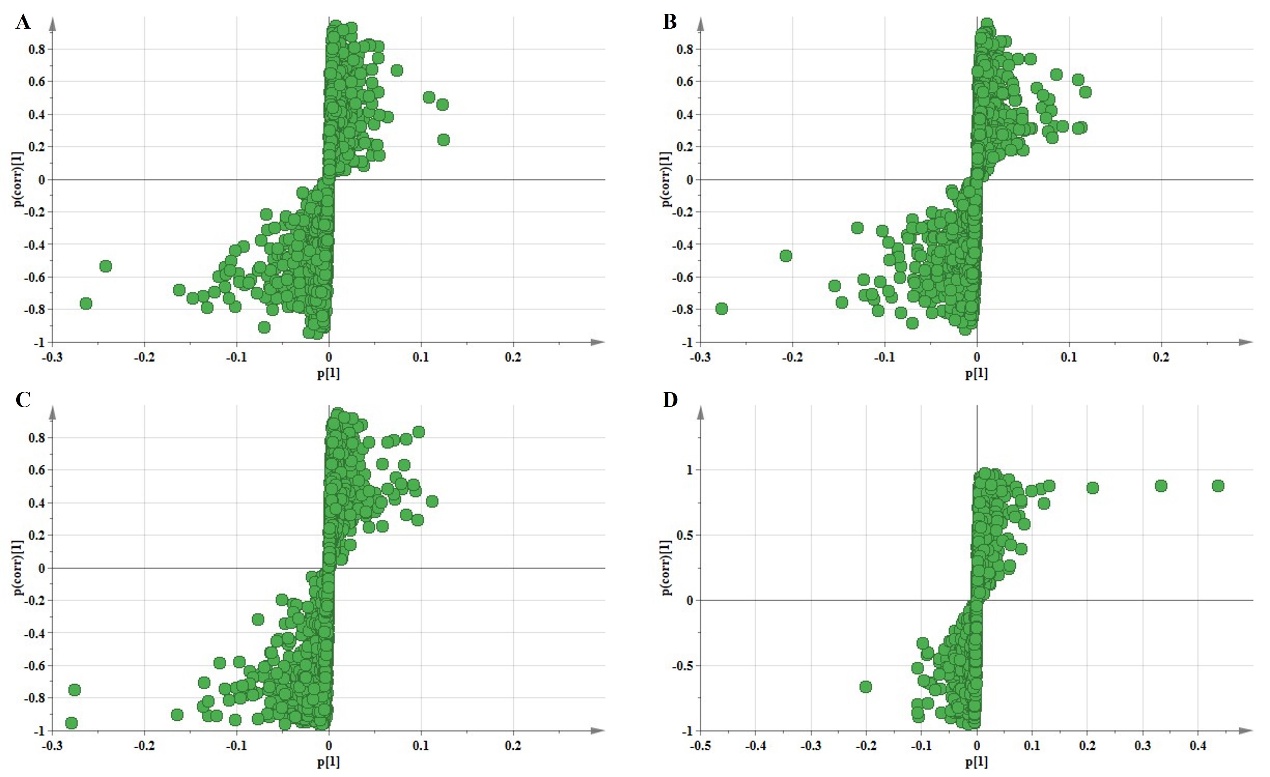


Supplementary Figure 10 S-plot score plot of ileocecal contents NEG from different samples in rats subjected to various intervention protocols

Note: A: LF vs Model; B: MF vs Model; C: HF vs Model; D: FIAC vs Model


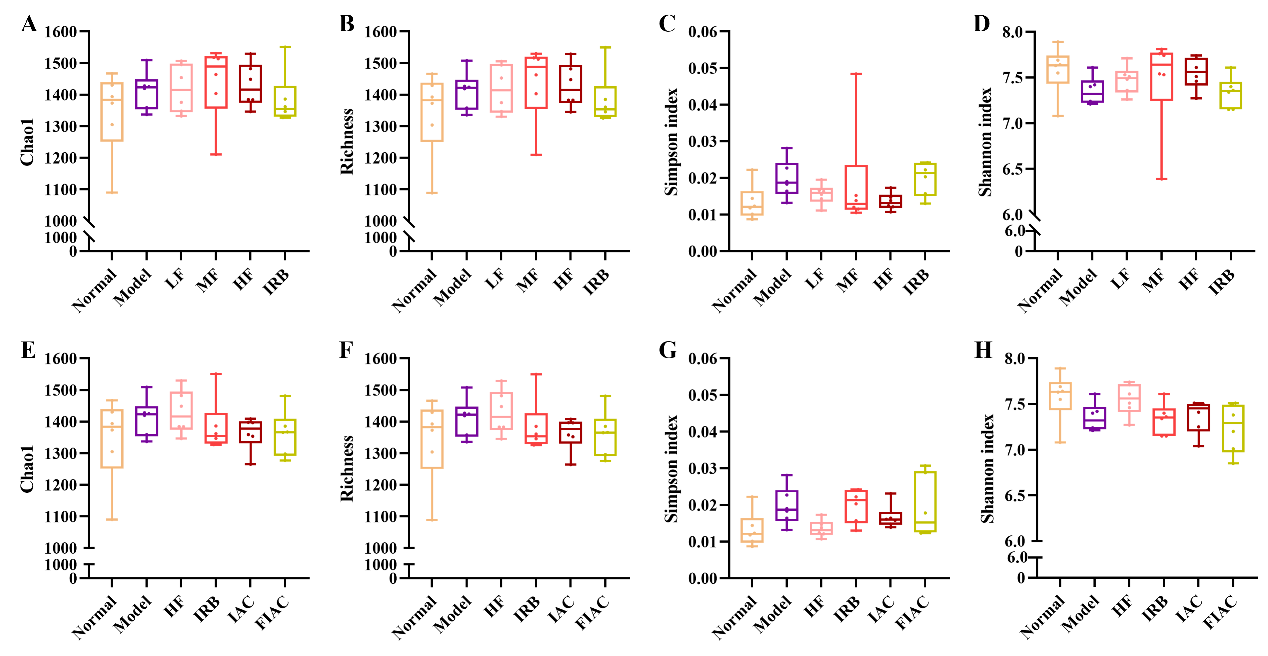


Supplementary Figure 11 Alpha diversity analysis of gut microbiota based on Chao, Ace, Shannon, and Simpson indices.

Note: A–D: Alpha diversity analysis of different concentrations of DOC; E–H: Alpha diversity analysis of DOC in combination with antihypertensive drugs.


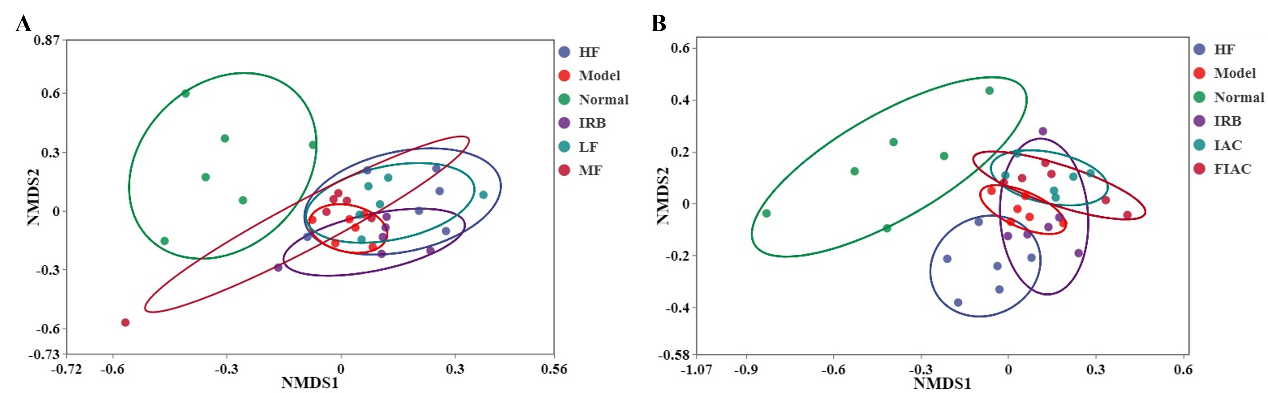


Supplementary Figure 12 Beta Diversity Analysis Based on NMDS

Note: (A) NMDS analysis of samples across different DOC concentrations; (B) NMDS analysis of samples treated with DOC in combination with antihypertensive drugs.


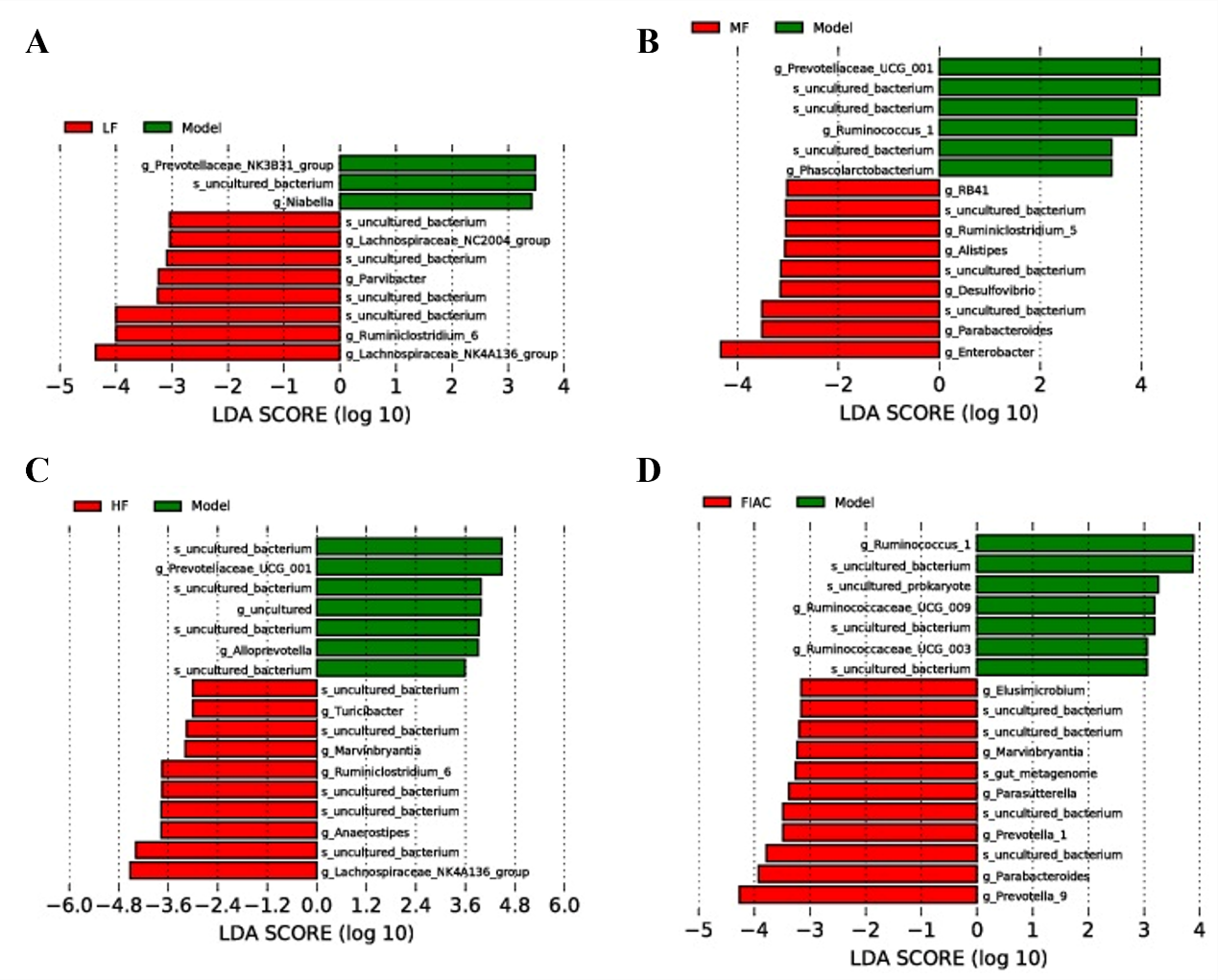


Supplementary Figure 13 LDA Score Plots Comparing Each Treatment Group with the Model Group

Note: A: LDA score plot comparing the MF group with the Model group; B: LDA score plot comparing the HF group with the Model group; C: LDA score plot comparing the integrated traditional Chinese and Western medicine group with the Model group.
