# Supplementary material for: A mechanistic study on the enhanced antihypertensive effects of a Dendrobium officinale compound combined with Western antihypertensive drugs in spontaneously hypertensive rats based on metabolomics and gut microbiota analysis
Source: Front Cell Dev Biol. 2026 Jun 15;14:1806395. doi: 10.3389/fcell.2026.1806395 (PMC13315239; doi:10.3389/fcell.2026.1806395)
Supplement: Supplementary file 2 [file Table1.doc]

Supplementary Table 1 The magnitude of blood pressure reduction in SHRs induced by various concentrations of DOC, as well as the combination of high-concentration DOC with conventional antihypertensive drugs

| Administration Time | Model | IRB | LF | MF | HF | IAC | FIAC |
| --- | --- | --- | --- | --- | --- | --- | --- |
| First administration at 3 hours post-dose | -1.6±5.27 | -23.58±7.61△△ | -11.11±3.66△△##◆◆▲▲ | -21.65±5.53△△ | -23.6±5.73△△ | -24.20±6.91△△◇◇ | -43.41±2.72△△ |
| First administration at 24 hours post-dose | 0.62±5.39 | -16.48±4.18△△ | -2.27±3.04##◆◆▲▲ | -15.58±5.41△△ | -17.1±3.87△△ | -20.31±10.25△ | -36.04±4.11△△ |
| At 3 hours on the 2nd week of dosing | -0.24±5.81 | -21.77±3.72△△ | -9.46±5.33△△##◆◆▲▲ | -21.25±5.52△△ | -21.62±2.67△△ | -22.24±7.16△△◇◇ | -41.06±7.55△△ |
| At 24 hours on the 2nd week of dosing | 0.43±3.33 | -22.12±8.27△△ | -1.22±3.53##◆◆▲▲ | -17.02±3.94△△ | -19.02±2△△ | -22.38±3.77△△◇◇ | -35.92±4.00△△ |
| At 3 hours on the 4th week of dosing | -0.74±3.9 | -22.93±2.86△△ | -10.77±3.58△△##◆◆▲▲ | -21.61±3.07△△ | -21.8±2.18△△ | -23.33±5.01△△◇◇ | -43.54±2.71△△ |
| At 24 hours on the 4th week of dosing | 1.9±3.7 | -21.47±2.75△△ | -1.59±3.84##◆◆▲▲ | -17.97±4.91△△ | -17.88±1.95△△ | -21.81±3.94△△◇◇ | -34.2±4.00△△ |
| At 3 hours on the 6th week of dosing | 0.66±2.55 | -22.08±2.9△△ | -11.56±2.06△△##◆◆▲▲ | -19.24±5.1△△ | -19.62±5.39△△ | -22.55±5.4△△◇◇ | -44.82±2.37△△ |
| At 24 hours on the 6th week of dosing | 0.22±2.21 | -20.34±3.87△△ | -1.97±3.09##◆◆▲▲ | -18.15±2.88△△ | -17.27±1.79△△ | -19.15±5.39△△◇◇ | -34.03±2.69△△ |

Note: Normal: Wistar rat group; Model: SHRs model group; IRB: treatment group administered with Irbesartan; LF: treatment group administered with 0.7 g/kg DOC (low concentration); MF: treatment group administered with 1.4 g/kg DOC (medium concentration); HF: treatment group administered with 2.8 g/kg DOC (high concentration); IAC: treatment group receiving combination therapy of Irbesartan and Amlodipine Besylate; FIAC: treatment group receiving high-dose DOC in combination with the two Western antihypertensive drugs. Comparison of each treatment group with the Model group: △: P < 0.05, △△: P <0.01. Comparison with the IRB group: ##: P < 0.01. Comparison with the HF group: ◆◆: P < 0.01; Comparison with the MF group: ▲▲: P< 0.01. Comparison with the FIAC group: ◇◇: P < 0.01.

Supplementary Table 2 Differential Metabolites Analysis of Rat Serum POS Samples: LF vs Model by OPLS-DA

| **NO.** | **Metabolites name** | **Molecular formula** | | **Quant mz** | **VIP** |
| --- | --- | --- | --- | --- | --- |
| **1** | Phenylacetonitrile | | C_8_H_7_N | 118.0651 | 1.7611 |
| **2** | Abrine | | C_12_H_14_N_2_O_2_ | 219.1131 | 1.75908 |
| **3** | L-Tyrosine | | C_9_H_11_NO_3_ | 182.081 | 1.64028 |
| **4** | L-Methionine | | C_5_H_11_NO_2_S | 150.0582 | 1.49008 |
| **5** | Citronellate | | C_10_H_18_O_2_ | 171.1379 | 1.46447 |
| **6** | 11-Dehydrocorticosterone | | C_21_H_28_O_4_ | 345.2063 | 1.42394 |
| **7** | 5-Aminopentanoate | | C_5_H_11_NO_2_ | 118.0862 | 1.39118 |
| **8** | O-Acetylcarnitine | | C_9_H_17_NO_4_ | 204.1229 | 1.3724 |
| **9** | Spermidine | | C_7_H_19_N_3_ | 146.1652 | 1.3446 |
| **10** | 11-Deoxycortisol | | C_21_H_30_O_4_ | 347.2215 | 1.3114 |
| **11** | Urate | | C_5_H_4_N_4_O_3_ | 169.0356 | 1.305 |
| **12** | L-Histidine | | C_6_H_9_N_3_O_2_ | 156.0768 | 1.24163 |
| **13** | L-Proline | | C_5_H_9_NO_2_ | 116.0706 | 1.14108 |
| **14** | gamma-Butyrolactone | | C_4_H_6_O_3_ | 87.044 | 1.13786 |
| **15** | L-Octanoylcarnitine | | C_15_H_29_NO_4_ | 288.2169 | 1.02782 |
| **16** | Tetrahydrocorticosterone | | C_21_H_34_O_4_ | 351.253 | 1.02695 |

Supplementary Table 3 Differential Metabolites Analysis of Rat Serum POS Samples: MF vs Model by OPLS-DA

| **NO.** | **Metabolites name** | **Molecular formula** | | **Quant mz** | **VIP** |
| --- | --- | --- | --- | --- | --- |
| **1** | Phenylacetonitrile | | C_8_H_7_N | 118.0651 | 1.53983 |
| **2** | Abrine | | C_12_H_14_N_2_O_2_ | 219.1131 | 1.43561 |
| **3** | L-Tyrosine | | C_9_H_1_NO_3_ | 182.081 | 1.43273 |
| **4** | Spermidine | | C_7_H_19_N_3_ | 146.1652 | 1.34026 |
| **5** | Citronellate | | C_10_H_18_O_2_ | 171.1379 | 1.33612 |
| **6** | L-Methionine | | C_5_H_11_NO_2_S | 150.0582 | 1.25368 |
| **7** | Progesterone | | C_21_H_30_O_2_ | 315.2319 | 1.17698 |
| **8** | 11-Dehydrocorticosterone | | C_21_H_28_O_4_ | 345.2063 | 1.08543 |
| **9** | 11beta,21-Dihydroxy-5beta-pregnane-3,20-dione | | C_21_H_28_O_4_ | 349.2376 | 1.05692 |
| **10** | Urate | | C_5_H_4_N_4_O_3_ | 169.0356 | 1.02605 |
| **11** | L-Arginine | | C_6_H_14_N_4_O_2_ | 175.1191 | 1.02301 |

Supplementary Table 4 Differential Metabolites Analysis of Rat Serum POS Samples: HF vs Model by OPLS-DA

| **NO.** | **Metabolites name** | **Molecular formula** | | **Quant mz** | **VIP** |
| --- | --- | --- | --- | --- | --- |
| **1** | L-Tyrosine | | C9H_11_NO_3_ | 182.081 | 1.63881 |
| **2** | Phenylacetonitrile | | C_8_H_7_N | 118.0651 | 1.54415 |
| **3** | Abrine | | C_12_H_14_N_2_O_2_ | 219.1131 | 1.51658 |
| **4** | L-Methionine | | C_5_H_11_NO_2_S | 150.0582 | 1.43065 |
| **5** | Spermidine | | C_7_H_19_N_3_ | 146.1652 | 1.42812 |
| **6** | 11-Deoxycortisol | | C_21_H_30_O_4_ | 347.2215 | 1.42145 |
| **7** | Citronellate | | C_10_H_18_O_2_ | 171.1379 | 1.40794 |
| **8** | L-Arginine | | C_6_H_14_N_4_O_2_ | 175.1191 | 1.35675 |
| **9** | Urate | | C_5_H_4_N_4_O_3_ | 169.0356 | 1.33435 |
| **10** | 2-Quinolinecarboxylic acid | | C_10_H_7_NO_2_ | 174.0553 | 1.2887 |
| **11** | Tetrahydrocorticosterone | | C_21_H_34_O_4_ | 351.253 | 1.23261 |
| **12** | 5-Aminopentanoate | | C_5_H_11_NO_2_ | 118.0862 | 1.15824 |
| **13** | Urea | | CH_4_N_2_O | 61.0395 | 1.13035 |
| **14** | Deoxycytidine | | C_9_H_13_N_3_O_4_ | 228.0983 | 1.11805 |
| **15** | L-Octanoylcarnitine | | C_15_H_29_NO_4_ | 288.2169 | 1.11196 |
| **16** | 3alpha,20alpha,21- Trihydroxy-5beta-pregnan-11- one | | C_21_H_34_O_4_ | 351.2529 | 1.06844 |

Supplementary Table 5 Differential Metabolites Analysis of Rat Serum POS Samples: FIAC vs Model by OPLS-DA

| **NO.** | **Metabolites name** | **Molecular formula** | | | **Quant mz** | | **VIP** |
| --- | --- | --- | --- | --- | --- | --- | --- |
| **1** | Urate | | C_5_H_4_N_4_O_3_ | 169.0356 | | 1.73189 | |
| **2** | Pregnenolone | | C_21_H_32_O_2_ | 317.2473 | | 1.61868 | |
| **3** | 2-Quinolinecarboxylic acid | | C_10_H_7_NO_2_ | 174.0553 | | 1.54226 | |
| **4** | Creatine | | C_4_H_9_N_3_O_2_ | 132.0766 | | 1.4388 | |
| **5** | Urea | | CH_4_N_2_O | 61.0395 | | 1.43638 | |
| **6** | L-Phenylalanine | | C_9_H_11_NO_2_ | 166.0862 | | 1.37545 | |
| **7** | Phenylacetonitrile | | C_8_H_7_N | 118.0651 | | 1.36429 | |
| **8** | L-Tyrosine | | C_9_H_11_NO_3_ | 182.081 | | 1.3218 | |
| **9** | 11-Dehydrocorticosterone | | C_21_H_28_O_4_ | 345.2063 | | 1.27516 | |
| **10** | 4-Methyl-2-oxopentanoate | | C_6_H_10_O_3_ | 131.0686 | | 1.01497 | |

Supplementary Table 6 Differential Metabolites Analysis of Rat Serum NEG Samples: LF vs Model by OPLS-DA

| **NO.** | **Metabolites name** | **Molecular formula** | | | **Quant mz** | | **VIP** |
| --- | --- | --- | --- | --- | --- | --- | --- |
| **1** | Taurine | | C_2_H_7_NO_3_S | 124.0073 | | 1.10195 | |
| **2** | Succinate | | C_4_H_6_O_4_ | 117.0196 | | 1.07807 | |
| **3** | Chenodeoxycholate | | C_24_H_40_O_4_ | 391.2855 | | 1.06797 | |

Supplementary Table 7 Differential Metabolites Analysis of Rat Serum NEG Samples: MF vs Model by OPLS-DA

| **NO.** | **Metabolites name** | **Molecular formula** | | | **Quant mz** | | **VIP** |
| --- | --- | --- | --- | --- | --- | --- | --- |
| **1** | Succinate | | C_4_H_6_O_4_ | 117.0196 | | 1.23789 | |
| **2** | D-Mannose | | C_6_H_12_O_6_ | 179.0562 | | 1.07403 | |

Supplementary Table 8 Differential Metabolites Analysis of Rat Serum NEG Samples: HF vs Model by OPLS-DA

| **NO.** | **Metabolites name** | **Molecular formula** | | | **Quant mz** | | **VIP** |
| --- | --- | --- | --- | --- | --- | --- | --- |
| **1** | D-Mannose | | C_6_H_12_O_6_ | 179.0562 | | 1.25937 | |
| **2** | Chenodeoxycholate | | C_24_H_40_O_4_ | 391.2855 | | 1.21533 | |
| **3** | Succinate | | C_4_H_6_O_4_ | 117.0196 | | 1.21281 | |

Supplementary Table 9 Differential Metabolites Analysis of Rat Serum NEG Samples: FIAC vs Model by OPLS-DA

| **NO.** | **Metabolites name** | **Molecular formula** | | | **Quant mz** | | **VIP** |
| --- | --- | --- | --- | --- | --- | --- | --- |
| **1** | L-Phenylalanine | | C_9_H_11_NO_2_ | 164.0715 | | 1.95944 | |
| **2** | Hyodeoxycholate | | C_24_H_40_O_4_ | 391.2856 | | 1.40303 | |
| **3** | Ascorbate | | C_6_H_8_O_6_ | 175.0252 | | 1.35483 | |
| **4** | Chenodeoxycholate | | C_24_H_40_O_4_ | 391.2855 | | 1.2799 | |
| **5** | Glycerone | | C_3_H_8_O_3_ | 89.0239 | | 1.11496 | |

Supplementary Table 10 Differential Metabolites Analysis of Rat Ileocecal Contents POS Samples: LF vs Model by OPLS-DA

| **NO.** | **Metabolites name** | **Molecular formula** | | **Quant mz** | **VIP** |
| --- | --- | --- | --- | --- | --- |
| **1** | Pregnenolone | | C_21_H_32_O_2_ | 317.2473 | 1.39133 |
| **2** | Urocortisol | | C_21_H_34_O_5_ | 367.2472 | 1.37226 |
| **3** | L-Proline | | C_5_H_9_NO_2_ | 116.0706 | 1.34796 |
| **4** | Ergosta-5,7,22,24(28)-tetraen-3beta-ol | | C_28_H_42_O | 395.3309 | 1.3273 |
| **5** | (24R,24(1)R)- Fucosterol epoxide | | C_29_H_48_O_2_ | 429.3723 | 1.32532 |
| **6** | Corticosterone | | C_21_H_30_O_4_ | 347.2218 | 1.29833 |
| **7** | Urocanate | | C_6_H_6_N_2_O_2_ | 139.0501 | 1.27267 |
| **8** | L-Carnitine | | C_7_H_15_NO_3_ | 162.1125 | 1.25822 |
| **9** | 11beta,17alpha,21-Trihydroxy-5beta-  pregnane-3,20-dione | | C_21_H_32_O_5_ | 365.2323 | 1.24639 |
| **10** | Androstenediol | | C_19_H_30_O_2_ | 291.2323 | 1.24122 |
| **11** | 2-Aminobut-2-enoate | | C_4_H_7_NO_2_ | 102.0552 | 1.23766 |
| **12** | 21-Hydroxy-5beta-pregnane-3,11,20- trione | | C_21_H_30_O_4_ | 347.2222 | 1.18555 |
| **13** | 3alpha,7alpha,12alpha-Trihydroxy-  5beta-cholestane | | C_27_H_48_O_3_ | 421.3662 | 1.1793 |
| **14** | L-Palmitoylcarnitine | | C_23_H_45_NO_4_ | 400.3419 | 1.13312 |
| **15** | Urocortisol | | C_21_H_34_O_5_ | 367.2482 | 1.11615 |
| **16** | 2-Quinolinecarboxylic acid | | C_10_H_7_NO_2_ | 174.0552 | 1.09883 |
| **17** | L-Phenylalanine | | C_9_H_11_NO_2_ | 166.0864 | 1.09815 |
| **18** | Benzyl 2-methyl-3-oxobutanoate | | C_12_H_14_O_3_ | 207.1019 | 1.09134 |
| **19** | Harman | | C_12_H_10_N_2_ | 183.0921 | 1.0853 |
| **20** | 3alpha,7alpha,12alpha,26-  Tetrahydroxy-5beta-cholestane | | C_27_H_48_O_4_ | 437.3622 | 1.08507 |
| **21** | alpha-Tocopherol | | C_29_H_50_O_2_ | 431.3875 | 1.07279 |
| **22** | L-Valine | | C_5_H_11_NO_2_ | 118.0862 | 1.07001 |
| **23** | 7alpha-  Hydroxydehydroepiandrosterone | | C_19_H_44_O_2_ | 305.2112 | 1.06966 |
| **24** | (25S)-26-Hydroxycholest-4-en-3-one | | C_27_H_44_O_2_ | 401.3409 | 1.06707 |

Supplementary Table 11 Differential Metabolites Analysis of Rat Ileocecal Contents POS Samples: MF vs Model by OPLS-DA

| **NO.** | **Metabolites name** | **Molecular formula** | | **Quant mz** | **VIP** |
| --- | --- | --- | --- | --- | --- |
| **1** | alpha-Tocopherol | | C29H50O2 | 431.3875 | 1.58331 |
| **2** | (24R,24(1)R)-Fucosterol epoxide | | C29H48O2 | 429.3723 | 1.51002 |
| **3** | Urocortisol | | C21H34O5 | 367.2472 | 1.35995 |
| **4** | 20-Hydroxy-3-oxopregn-4-en-21-al | | C21H30O3 | 331.2244 | 1.33745 |
| **5** | 11beta,17alpha,21-  Trihydroxypregnenolone | | C21H32O5 | 365.2321 | 1.32722 |
| **6** | Ergosta-5,7,22,24(28)-tetraen-  3beta-ol | | C28H42O | 395.3309 | 1.30962 |
| **7** | Pregnenolone | | C21H32O2 | 317.2473 | 1.27346 |
| **8** | L-Phenylalanine | | C9H11NO2 | 166.0864 | 1.26006 |
| **9** | Androstenediol | | C19H30O2 | 291.2323 | 1.26002 |
| **10** | D-Phenylalanine | | C9H11NO2 | 166.0864 | 1.23354 |
| **11** | 3alpha,7alpha,12alpha,26- Tetrahydroxy-5beta-cholestane | | C27H48O4 | 437.3622 | 1.20921 |
| **12** | Pregnanediol | | C21H36O2 | 321.2788 | 1.20746 |
| **13** | 3alpha,7alpha,12alpha- Trihydroxy-5beta-cholestane | | C27H48O3 | 421.3662 | 1.18905 |
| **14** | 11beta,17alpha,21-Trihydroxy- 5beta-pregnane-3,20-dione | | C21H32O5 | 365.2323 | 1.12343 |
| **15** | L-Proline | | C5H9NO2 | 116.0706 | 1.11094 |
| **16** | Creatine | | C4H9N3O2 | 132.0767 | 1.10996 |
| **17** | L-Valine | | C_5_H_11_NO_2_ | 118.0862 | 1.0645 |
| **18** | L-Leucine | | C6H13NO2 | 132.102 | 1.05634 |
| **19** | Sinapyl alcohol | | C11H14O4 | 211.0943 | 1.01372 |
| **20** | Lactose | | C12H22O11 | 343.1239 | 1.0042 |

Supplementary Table 12 Differential Metabolites Analysis of Rat Ileocecal Contents POS Samples: HF vs Model by OPLS-DA

| **NO.** | **Metabolites name** | **Molecular formula** | **Quant mz** | **VIP** |
| --- | --- | --- | --- | --- |
| **1** | (24R,24(1)R)- Fucosterol epoxide | C_29_H_48_O_2_ | 429.3723 | 1.50926 |
| **2** | Ergosta-5,7,22,24(28)-tetraen-3beta- ol | C_28_H_42_O | 395.3309 | 1.49137 |
| **3** | Allotetrahydrodeoxycorticosterone | C_21_H_34_O_3_ | 335.2577 | 1.42876 |
| **4** | Pregnenolone | C_21_H_32_O_2_ | 317.2473 | 1.40605 |
| **5** | Urocortisol | C_21_H_34_O_5_ | 367.2472 | 1.39295 |
| **6** | 11beta,17alpha,21- Trihydroxypregnenolone | C_21_H_32_O_5_ | 365.2321 | 1.37462 |
| **7** | 5-Acetamidopentanoate | C_7_H_13_NO_3_ | 160.0968 | 1.36704 |
| **8** | alpha-Tocopherol | C_29_H_50_O_2_ | 431.3875 | 1.36352 |
| **9** | 7alpha- Hydroxydehydroepiandrosterone | C_19_H_28_O_3_ | 305.2112 | 1.34196 |
| **10** | Benzyl 2-methyl-3-oxobutanoate | C_12_H_14_O_3_ | 207.1019 | 1.28258 |
| **11** | 20-Hydroxy-3-oxopregn-4-en-21-al | C_21_H_30_O_3_ | 331.2244 | 1.25533 |
| **12** | Corticosterone | C_21_H_30_O_4_ | 347.2218 | 1.23364 |
| **13** | 21-Hydroxy-5beta-pregnane-3,11,20- trione | C_21_H_30_O_4_ | 347.2222 | 1.22385 |
| **14** | 20alpha,22beta-Dihydroxycholesterol | C_27_H_46_O_3_ | 419.3524 | 1.21493 |
| **15** | 3alpha,7alpha,12alpha-Trihydroxy-5beta-cholestane | C_27_H_48_O_3_ | 421.3662 | 1.21182 |
| **16** | 11beta,17alpha,21-Trihydroxy-5beta- pregnane-3,20-dione | C_21_H_32_O_5_ | 365.2323 | 1.16598 |
| **17** | (25S)-26-Hydroxycholest-4-en-3-one | C_27_H_44_O_2_ | 401.3409 | 1.16092 |
| **18** | Androstenediol | C_19_H_30_O_2_ | 291.2323 | 1.15204 |
| **19** | 17alpha,21-Dihydroxypregnenolone | C_21_H_32_O_4_ | 349.2374 | 1.13573 |
| **20** | Harman | C_12_H_10_N_2_ | 183.0921 | 1.12355 |
| **21** | 3alpha,7alpha,12alpha,26- Tetrahydroxy-5beta-cholestane | C_27_H_48_O_4_ | 437.3622 | 1.1018 |
| **22** | L-Proline | C_5_H_9_NO_2_ | 116.0706 | 1.08971 |
| **23** | 17alpha-Hydroxypregnenolone | C_21_H_32_O_3_ | 333.2422 | 1.04751 |
| **24** | Urocanate | C_6_H_6_N_2_O_2_ | 139.0501 | 1.0289 |

Supplementary Table 13 Differential Metabolites Analysis of Rat Ileocecal Contents POS Samples: FIAC vs Model by OPLS-DA

| **NO.** | **Metabolites name** | **Molecular formula** | **Quant mz** | **VIP** |
| --- | --- | --- | --- | --- |
| **1** | Urocortisol | C_21_H_34_O_5_ | 367.2472 | 1.36538 |
| **2** | Ergosta-5,7,22,24(28)-tetraen-3beta- ol | C_28_H_42_O | 395.3312 | 1.31636 |
| **3** | 4,4-Dimethyl-5alpha-cholesta-8,14,24-trien-3beta-ol | C_29_H_46_O | 411.3617 | 1.30075 |
| **4** | 11beta,17alpha,21- Trihydroxypregnenolone | C_21_H_32_O_5_ | 365.2321 | 1.2764 |
| **5** | 3alpha,7alpha-Dihydroxy-5beta- cholestanate | C_27_H_46_O_4_ | 435.3468 | 1.23435 |
| **6** | Pregnenolone | C_21_H_32_O_2_ | 317.2473 | 1.2102 |
| **7** | (25S)-26-Hydroxycholest-4-en-3- one | C_27_H_44_O_2_ | 401.3409 | 1.20617 |
| **8** | L-Tyrosine | C_9_H_11_NO_3_ | 182.0812 | 1.19509 |
| **9** | L-Proline | C_5_H_9_NO_2_ | 116.0706 | 1.19022 |
| **10** | Androstenediol | C_19_H_30_O_2_ | 291.2323 | 1.16827 |
| **11** | 3alpha,7alpha,12alpha-Trihydroxy-5beta-cholestane | C_27_H_48_O_3_ | 421.3662 | 1.15799 |
| **12** | 3alpha,7alpha,12alpha,26- Tetrahydroxy-5beta-cholestane | C_27_H_48_O_4_ | 437.3622 | 1.14827 |
| **13** | 11beta,17alpha,21-Trihydroxy- 5beta-pregnane-3,20-dione | C_21_H_32_O_5_ | 365.2323 | 1.10316 |
| **14** | Corticosterone | C_21_H_30_O_4_ | 347.2218 | 1.10312 |
| **15** | Urocanate | C_6_H_6_N_2_O_2_ | 139.0501 | 1.10277 |
| **16** | Sinapyl alcohol | C_11_H_14_O_4_ | 211.0943 | 1.10235 |
| **17** | 17alpha-Hydroxypregnenolone | C_21_H_32_O_3_ | 333.2422 | 1.0734 |
| **18** | 17alpha,21-Dihydroxypregnenolone | C_21_H_32_O_4_ | 349.2374 | 1.06346 |
| **19** | 17alpha,21-Dihydroxy-5beta- pregnane-3,11,20-trione | C_21_H_30_O_5_ | 363.2172 | 1.03649 |
| **20** | 7alpha-Hydroxydehydroepiandrosterone | C_19_H_28_O_3_ | 305.2112 | 1.03639 |
| **21** | 22alpha-Hydroxy-campest-4-en-3- one | C_28_H_46_O_2_ | 415.3573 | 1.03328 |
| **22** | D-Phenylalanine | C_9_H_11_NO_2_ | 166.0864 | 1.01922 |
| **23** | Cortolone | C_21_H_34_O_5_ | 367.2482 | 1.01912 |

Supplementary Table 14 Differential Metabolites Analysis of Rat Ileocecal Contents NEG Samples: LF vs Model by OPLS-DA

| **NO.** | **Metabolites name** | **Molecular formula** | **Quant mz** | **VIP** |
| --- | --- | --- | --- | --- |
| **1** | 3alpha,20alpha,21-Trihydroxy- 5beta-pregnan-11-one | C_21_H_34_O_4_ | 349.2381 | 1.44933 |
| **2** | Oleanolic acid | C_30_H_48_O_3_ | 455.3532 | 1.2439 |
| **3** | Succinate | C_4_H_6_O_4_ | 117.0194 | 1.21282 |
| **4** | L-Glutamate | C_5_H_9_NO_4_ | 146.0459 | 1.20909 |
| **5** | 3alpha,7alpha-Dihydroxy-12-oxo-5beta-cholanate | C_24_H_38_O_5_ | 405.2646 | 1.11952 |
| **6** | Lithocholic acid | C_24_H_40_O_3_ | 375.2904 | 1.11706 |
| **7** | Tetrahydrocorticosterone | C_21_H_34_O_4_ | 349.2389 | 1.06001 |

Supplementary Table 15 Differential Metabolites Analysis of Rat Ileocecal Contents NEG Samples: MF vs Model by OPLS-DA

| **NO.** | **Metabolites name** | **Molecular formula** | **Quant mz** | **VIP** |
| --- | --- | --- | --- | --- |
| **1** | 3alpha,20alpha,21-Trihydroxy- 5beta-pregnan-11-one | C_21_H_34_O_4_ | 349.2381 | 1.55532 |
| **2** | Lithocholic acid | C_24_H_40_O_3_ | 375.2904 | 1.24978 |
| **3** | Tetrahydrocorticosterone | C_21_H_34_O_4_ | 349.2389 | 1.23471 |
| **4** | 3alpha,7alpha-Dihydroxy-12- oxo-5beta-cholanate | C_24_H_38_O_5_ | 405.2646 | 1.19213 |
| **5** | L-Glutamate | C_5_H_9_NO_4_ | 146.0459 | 1.18199 |
| **6** | Hypoxanthine | C_5_H_4_N_4_O | 135.0312 | 1.06758 |
| **7** | Succinate | C_4_H_6_O_4_ | 117.0194 | 1.03979 |
| **8** | Oleanolic acid | C_30_H_48_O_3_ | 455.3532 | 1.03253 |

Supplementary Table 16 Differential Metabolites Analysis of Rat Ileocecal Contents NEG Samples: HF vs Model by OPLS-DA

| **NO.** | **Metabolites name** | **Molecular formula** | **Quant mz** | **VIP** |
| --- | --- | --- | --- | --- |
| **1** | 3alpha,20alpha,21-Trihydroxy- 5beta-pregnan-11-one | C_21_H_34_O_4_ | 349.2381 | 1.45497 |
| **2** | Tetrahydrocorticosterone | C_21_H_34_O_4_ | 349.2389 | 1.40382 |
| **3** | Lithocholic acid | C_24_H_40_O_3_ | 375.2904 | 1.2329 |
| **4** | L-Glutamate | C_5_H_9_NO_4_ | 146.0459 | 1.21553 |
| **5** | 3alpha,7alpha-Dihydroxy-12-oxo-5beta-cholanate | C_24_H_38_O_5_ | 405.2646 | 1.09829 |
| **6** | Oleanolic acid | C_30_H_48_O_3_ | 455.3532 | 1.03184 |

Supplementary Table 17 Differential Metabolites Analysis of Rat Ileocecal Contents NEG Samples: FIAC vs Model by OPLS-DA

| **NO.** | **Metabolites name** | **Molecular formula** | **Quant mz** | **VIP** |
| --- | --- | --- | --- | --- |
| **1** | Tetrahydrocorticosterone | C_21_H_34_O_4_ | 349.2389 | 1.49057 |
| **2** | Oleanolic acid | C_30_H_48_O_3_ | 455.3532 | 1.44319 |
| **3** | Lithocholic acid | C_24_H_40_O_3_ | 375.2904 | 1.24573 |
| **4** | 16-Hydroxypalmitate L-Glutamate | C_16_H_32_O_3_ | 271.2287 | 1.23186 |
| **5** | 3alpha,7alpha-Dihydroxy-12- oxo-5beta-cholanate | C_5_H_9_NO_4_ | 146.0459 | 1.07168 |
| **6** | Cholic acid | C_24_H_38_O_5_ | 405.2646 | 1.03639 |
| **7** | Oleanolic acid | C_24_H_4_O_5_ | 407.2799 | 1.01129 |
